# The Stable Gastric Pentadecapeptide BPC 157 Pleiotropic Beneficial Activity and Its Possible Relations with Neurotransmitter Activity

**DOI:** 10.3390/ph17040461

**Published:** 2024-04-03

**Authors:** Predrag Sikiric, Alenka Boban Blagaic, Sanja Strbe, Lidija Beketic Oreskovic, Ivana Oreskovic, Suncana Sikiric, Mario Staresinic, Marko Sever, Antonio Kokot, Ivana Jurjevic, Danijel Matek, Luka Coric, Ivan Krezic, Ante Tvrdeic, Kresimir Luetic, Lovorka Batelja Vuletic, Predrag Pavic, Tomislav Mestrovic, Ivica Sjekavica, Anita Skrtic, Sven Seiwerth

**Affiliations:** 1 Department of Pharmacology, School of Medicine, University of Zagreb, 10000 Zagreb, Croatia; abblagaic@mef.hr (A.B.B.); strbes@gmail.com (S.S.); lidijabeketicoreskovic@gmail.com (L.B.O.); psikiric@gmail.com (I.O.); suncanasikiric@gmail.com (S.S.); ravnateljstvo@kb-merkur.hr (M.S.); dr.sever.marko@gmail.com (M.S.); antonio.kokot@mefos.hr (A.K.); ivana.jurjevic@mef.hr (I.J.); dmatek@gmail.com (D.M.); luka.coric3105@gmail.com (L.C.); ivankrezic94@gmail.com (I.K.); ante.tvrdeic@mef.hr (A.T.); kluetic@yahoo.com (K.L.); lbatelja@mef.hr (L.B.V.); p.d.pavic@gmail.com (P.P.); mestrovic.tomislav@gmail.com (T.M.); ivica.sjekavica@zg.t-com.hr (I.S.); sven.seiwerth@mef.hr (S.S.); 2 Department of Pathology, School of Medicine, University of Zagreb, 10000 Zagreb, Croatia; 3 Department of Surgery, School of Medicine, University of Zagreb, 10000 Zagreb, Croatia; 4 Department of Anatomy and Neuroscience, School of Medicine, J.J. Strossmayer University of Osijek, 31000 Osijek, Croatia; 5 Department of Diagnostic and Interventional Radiology, Sestre Milosrdnice University Hospital Center, 10000 Zagreb, Croatia

**Keywords:** stable gastric pentadecapeptide BPC 157, pleiotropic beneficial activity, cytoprotection, neurotransmitter, occlusion/occlusion-like syndrome, VEGF

## Abstract

We highlight the particular aspects of the stable gastric pentadecapeptide BPC 157 pleiotropic beneficial activity (not destroyed in human gastric juice, native and stable in human gastric juice, as a cytoprotection mediator holds a response specifically related to preventing or recovering damage as such) and its possible relations with neurotransmitter activity. We attempt to resolve the shortage of the pleiotropic beneficial effects of BPC 157, given the general standard neurotransmitter criteria, in classic terms. We substitute the lack of direct conclusive evidence (i.e., production within the neuron or present in it as a precursor molecule, released eliciting a response on the receptor on the target cells on neurons and being removed from the site of action once its signaling role is complete). This can be a network of interconnected evidence, previously envisaged in the implementation of the cytoprotection effects, consistent beneficial particular evidence that BPC 157 therapy counteracts dopamine, serotonin, glutamate, GABA, adrenalin/noradrenalin, acetylcholine, and NO-system disturbances. This specifically includes counteraction of those disturbances related to their receptors, both blockade and over-activity, destruction, depletion, tolerance, sensitization, and channel disturbances counteraction. Likewise, BPC 157 activates particular receptors (i.e., VGEF and growth hormone). Furthermore, close BPC 157/NO-system relations with the gasotransmitters crossing the cell membrane and acting directly on molecules inside the cell may envisage particular interactions with receptors on the plasma membrane of their target cells. Finally, there is nerve-muscle relation in various muscle disturbance counteractions, and nerve-nerve relation in various encephalopathies counteraction, which is also exemplified specifically by the BPC 157 therapy application.

## 1. Introduction

*The stable gastric pentadecapeptide BPC 157 pleiotropic beneficial activity and its possible relations with neurotransmitter activity*.

This review highlights the particular aspects of the stable gastric pentadecapeptide BPC 157 pleiotropic beneficial activity [1,2,3,4,5,6,7,8,9,10,11] and its possible relations with neurotransmitters’ activity [12,13,14] so far not emphasized.

There, the stable gastric pentadecapeptide BPC 157 studies, already largely reviewed [1,2,3,4,5,6,7,8,9,10,11], share some major worth-mentioning points given its development in the early 1990s [15] as a late outbreak of the already advanced, but not fully implemented, cytoprotection concept. Note that the cytoprotection concept, as originated by Robert [16,17] and Szabo [18,19,20,21], is commonly regarded as a breakthrough in gastroenterology [22,23,24,25,26].

Presently, BPC 157 therapy has still limited but encouraging clinical evidence (no toxicity in clinical trials (i.e., effective in ulcerative colitis, phase II) and toxicology studies without lethal dose (LD1)), and much more should be further achieved [1,2,3,4,5,6,7,8,9,10,11]. On the other hand, there is additional supportive evidence for the pleiotropic beneficial effect that can largely overwhelm possible limitations. Given the cytoprotection concept born in the stomach (innate prevention of the stomach epithelial [16,17] and endothelial [18,19,20,21] cell necrosis that should be extended to other tissue healing), BPC 157, native and stable in human gastric juice can be a cytoprotection mediator as it is not destroyed in human gastric juice for more than 24 h [1,2,3,4,5,6,7,8,9,10,11,27,28,29,30]. Worth mentioning, as Selye’s stress concept (Selye’s stress response against various noxious agents to reestablish homeostasis) [31,32] gave birth years later to Robert’s cytoprotection concept [16,17] (for review see, i.e., [33]), the cytoprotection concept holds a response that is specifically related to prevent or recover damage as such. Thereby, pleiotropic effects by cytoprotection agents against various noxious agents and procedures should likely be achieved by concept implementation [16,17,18,19,20,21].

BPC 157 therapy consistently holds µg-ng dose range applied alone without carrier addition and has no sequence homology with known gut peptides [1,2,3,4,5,6,7,8,9,10,11,15,27,28,29,30,33]. This ascertains its essential advantage in peptides background that the peptide’s background can be easily combined with cytoprotection pleiotropic beneficial effect and the obtained effects as proof that it avoids practical and theoretical limitations that impede peptides from showing undisputed activity (for review, see [10]). Namely, as easily destroyed, standard growth peptidergic factors, while endogenously present, commonly request the addition of various carriers (i.e., peptide+carrier(s) complex) to show the needed effect when applied [10]. Thereby, they can have uncertain activity attribution providing the given complex, peptide+carrier(s) (peptide, carrier, or peptide+carrier(s)) [10], essential limiting activity point, which is largely underestimated. Contrarily, there is easy application of BPC 157 using whatever way of application (parenteral or per-oral); BPC 157 as stable gastric pentadecapeptide has an undisputed therapy practical application and wide interconnected activity that can be regularly seen and consistently ascribed to its own activity [1,2,3,4,5,6,7,8,9,10,11,15,27,28,29,30,33].

Commonly, a neurotransmitter is considered to be a signaling molecule secreted by a neuron to affect another cell across a synapse [12,13,14]. The cell receiving the signal, or target cell, may be another neuron, but it could also be a gland or muscle cell [12,13,14]. It is also commonly acknowledged that through synaptic transmission (also known as neurotransmission), the CNS can control smooth, skeletal, and cardiac muscles, bodily secretions, and organ functions [12,13,14]. Thus, the essential significance goes to each of the substances designated to be a neurotransmitter, and thereby, the pleiotropic significance of the acknowledged neurotransmitters such as acetylcholine, histamine, dopamine, glutamine, glycine, GABA, noradrenaline, and adrenaline [12,13,14]. In addition, over 100 neuroactive peptides (i.e., many of these are co-released along with a small-molecule) transmitters have been found and are commonly thought to have essential functions in controlling essential systems such as reproduction, development, growth, energy homeostasis, cardiovascular activity, and stress response, thus animal physiology in general [34,35]. Given the activation of receptors as the only direct action of a neurotransmitter, beta-endorphin is a relatively well-known example of a peptide neurotransmitter primary transmitter at a synapse having highly specific interactions with opioid receptors in the central nervous system [34,35]. However, the gasotransmitters are not stored in synaptic vesicles and may carry messages from the postsynaptic neuron to the presynaptic neuron [36]. Rather than interacting with receptors on the plasma membrane of their target cells, the gasotransmitters (soluble gases such as nitric oxide and carbon monoxide, as unconventional neurotransmitters) can cross the cell membrane and act directly on molecules inside the cell [36].

Conceptually, in the implementation of the cytoprotection concept (huge pleiotropic beneficial effect of the cytoprotective agent [16,17,18,19,20,21]) for BPC 157 therapy effects, seen from the original viewpoint of the gut peptides’ significance and brain relation [1], we reviewed a wide beneficial effect, both peripherally and centrally, as essential for the harmony of the brain–gut and gut–brain axes’ function. There, the favorable stable gastric pentadecapeptide BPC 157 evidence in the brain–gut and gut–brain axes’ function might convey a particular interconnected network [1,37].

From the periphery, the immediate defensive response can be the stable gastric pentadecapeptide BPC 157 therapy [1,2,3,4,5,6,7,8,9,10,11,15,27,28,29,30,33], peripherally and centrally. Native and stable in human gastric juice, BPC 157 as a cytoprotection mediator can be easily applied, including via the per-oral route [1,2,3,4,5,6,7,8,9,10,11,15,27,28,29,30,33,37]. This can be a particular conceptual point, as cytoprotection represents a general concept of overall significance. The concept born by Robert (epithelium) [16,17] and Szabo (endothelium) [18,19,20,21] holds stomach integrity maintenance that should be translated to other organ healing as well by cytoprotective agents’ application [16,17,18,19,20,21]. Thus, as a hormone-like activity, BPC 157 can be released into the circulation and sent to distant organs by complex biological processes to regulate physiology and behavior. A study (^3^H-PL-10.1.AK-15. Pharmacokinetics in the rat after single oral administration. Istituto di Ricerche Biomediche “A. Marxer”, RBM: 3 May 1996) revealed a half-life of 66 h in male rats and 69 h in female rats. Thereby, based on the obtained beneficial evidence, we claimed that with BPC 157 therapy, cytoprotection [26,27,28,29,30] as a particular vascular effect [26,27,28,29,30], wound healing [6,7,8,9,10], and neuroprotection [1,5,37] combine the principle of upgrading minor vessels [26,27]. By BPC 157 therapy, the principle of upgrading minor vessels by prompt activation of the vascular collaterals (i.e., azygos vein direct blood flow delivery) is commonly operative to counteract the occlusion/occlusion-like syndrome, major vascular and multiorgan failure peripherally and centrally induced in rats [26,27]. These were induced with permanent major vessel occlusions, central [38,39] and peripheral [40,41,42,43,44,45,46], similar noxious procedures [47,48,49,50], and agents’ applications [51,52,53,54,55,56]. These all severely affect endothelium function.

Centrally [1], behavioral data provided anxiolytic [57,58,59], anticonvulsive [15,58,60,61,62,63,64], antidepressant effect [65,66,67], counteraction of catalepsy [68,69,70], and counteraction of positive and negative schizophrenia symptoms models [59,70]. Such a large range of mutually supporting behavioral findings gives rise to interaction with the main systems in a particular but well-orchestrated way (see Chapters Dopamine, Serotonin, Glutamate, GABA, Acetylcholine, Adrenaline/Noradrenaline and NO).

Muscle healing and function recovery [3] (i.e., with direct injury, transected [71], crushed [72,73], denervated muscle [74], and myotendinous junction [75]) also appeared as the therapeutic effects of BPC 157 on the various muscle disabilities of a multitude of causes [3] (see Chapter Nerve-Muscle). Illustratively, these prime lesions, both peripheral and central, were also ameliorated. These were counteracted/attenuated muscle disabilities induced by spinal cord injury [76,77], stroke [38], concussive brain trauma [78], nerve transection [79], and neurotoxin application (1-methyl-4-phenyl-1,2,3,6-tetrahydropyridine (MPTP) and cuprizone, mimicking Parkinson’s disease in mice and multiple sclerosis in rats) [69,80]. Likewise, counteracted/attenuated were both local and systemic muscle disabilities following muscle relaxant application (depolarizing neuromuscular blocker [81] and non-depolarizing neuromuscular blocker (report in preparation)). Additionally, counteracted/attenuated were severe muscle disturbances in rats with abdominal aorta occlusion [82], tumor cachexia (severe muscle wasting) [11], and different electrolyte disturbances [54,83,84,85]. Counteracted/attenuated were neuroleptics (i.e., catalepsy) [68,70], L-arginine (i.e., prolonged miosis) [86], N(G)-nitro-L-arginine methyl ester (L-NAME) (prolonged miosis, catalepsy) [70,86], and alcohol acute and chronic intoxication [63,64].

Disabling heart failure recovery as a whole [4] (myocardial infarction [53], heart failure [87], pulmonary hypertension [88], arrhythmias [40,41,42,47,48,49,50,51,52,53,54,55,56,60,81,89,90,91], and thrombosis presentation [40,41,42,43,47,48,49,50,51,52,53,54,55,56]) was reviewed [3,4]. Smooth muscle function recovery was also reviewed [3,4] (i.e., sphincter dysfunction (pupil [86], lower esophageal and pyloric sphincter [92,93,94,95,96,97,98,99], urethral sphincter [100,101,102], intestinal adaptation, and lesion recovery [103,104]). It was claimed that with BPC 157 therapy, these existed as a multimodal muscle axis control on muscle function and healing as a function of the brain–gut axis and gut–brain axis as a whole [1,3,4].

Finally, for encephalopathies therapy (see Chapter Nerve-Nerve), acting simultaneously in both the periphery and central nervous system, BPC 157 counteracted various encephalopathies and stomach and liver lesions in non-steroidal anti-inflammatory drugs (NSAIDs)-rats [61,105,106,107,108,109] and insulin-rats [62]. Noteworthy, these were recently combined with the mentioned evidence that BPC 157 therapy by rapidly activated collateral pathways (azygos vein direct blood flow delivery) goes as a particular therapy effect, as counteracted the vascular and multiorgan failure concomitant to various major vessel occlusion and similar various noxious procedures and various agents’ application [40,41,42,43,47,48,49,50,51,52,53,54,55,56]. This specifically reversed the initiated multicausal noxious circuit of the occlusion/occlusion-like syndrome as a general effect. Severe intracranial (superior sagittal sinus) hypertension, portal and caval hypertension, and aortal hypotension were attenuated/eliminated. Counteracted were the severe lesions and hemorrhage in the brain, lungs, liver, kidney, and gastrointestinal tract. In particular, progressing thrombosis, both peripherally and centrally, and heart arrhythmias and infarction that would consistently occur were fully counteracted and/or almost annihilated [40,41,42,43,47,48,49,50,51,52,53,54,55,56]. With these capabilities (already advanced Virchow triad circumstances were counteracted), BPC 157 therapy is effective in conditions of ischemia [40,41,42,43,47,49,50,51,52,53,54,55,56] and in conditions of reperfusion [38,48,76,77,110].

Consequently, these include also eye therapy; BPC 157 therapy can recover glaucomatous rats, normalize intraocular pressure, maintain retinal integrity, recover pupil function, recover retinal ischemia, and corneal injuries (i.e., maintain transparency after complete corneal abrasion, corneal ulceration, and counteract dry eye after lacrimal gland removal or corneal insensitivity) [2,45,86,111,112,113,114]. 

All these points may be supported by its special interaction with various molecular pathways [8,11,75,115,116,117,118,119,120,121,122,123], especially with the nitric oxide (NO)-system [121,122,123,124,125,126,127,128,129,130] as a whole. This implies a wide therapy counteracting potential (NO-release, NO-synthase (NOS)-inhibition (L-NAME), NOS-over-activity (L-arginine), NO-system immobilization (L-NAME+L-arginine), i.e., in hypertension, hypotension, and thrombocytes function (without affecting coagulation cascade) [124,125,126,127,128,129,130,131], signaling pathways controlling vasomotor tone [121,122,123] (VEGFR2-Akt-eNOS and Src-Caveolin-1-eNOS)). This also includes counteraction of anaphylactoid reaction [132]. It also acts as a free radical scavenger [8,11,75,110,133,134,135] in vascular occlusion/occlusion-like failure studies, in particular [40,41,42,43,47,48,49,50,51,52,53,54,55,56], associated with its function as a stabilizer of cellular junction [11], leading to the significantly mitigated leaky gut syndrome [11]. There, in the vessel wall, the rapid change in the lipid contents and protein secondary structure conformation, produced instantly via BPC 157 therapy [136] (Fourier transform infrared spectroscopy), supported the vessel function even in the worst circumstances. 

Possibly, the similar beneficial effects in other species (i.e., birds [137] and insects [138,139,140]) may suggest that BPC 157 may also have an extended regulatory physiologic role in bodily functions. 

Initially, it was immunohistochemically demonstrated in the rat stomach as well as in the brain [15]. A further study [10] used human BPC oligonucleotide probes and a specific BPC 157 polyclonal antibody to analyze BPC 157 expression and synthesis in human fetal and adult tissues. Northern blot hybridization revealed two mRNA species of 3 and 1.8 kb. Larger mRNA size was more pronounced in adults, while equal quantities of both mRNA species were detected in fetal tissues. High levels of BPC mRNA were found in adult gastrointestinal epithelium. By in situ hybridization and immunostaining, BPC 157 was found in gastrointestinal mucosa, lung bronchial epithelium, epidermal layer of the skin, and kidney glomeruli [10]. Thereby, it was suggested that in addition to BPC being isolated from gastric juice and probably primarily acting in the gastrointestinal system, it may have additional regulatory roles in the function of the human lung, kidney, and skin [10].

To conclude, for the suggested further BPC 157 therapy applications, the strength of evidence of its pleiotropic beneficial effects of therapy should be reviewed from the viewpoint of the possible neurotransmitter activity. Note, in general, since the beginning, the concept of cytoprotection did not consider the neurotransmitter activity as the basis for the cytoprotective effects of the implemented agents (i.e., prostaglandins [16,17], sulfhydryl [141], somatostatin [142], dopamine [143]). Although a possible neurotransmitter role was claimed, i.e., for prostaglandins [144,145,146,147] and somatostatin [148,149,150], this was not combined with the cytoprotective effect and possible pleiotropic effect (organoprotection). The central application of neurotensin as a neurotransmitter was a central basis of its cytoprotective effect but was not further extended [151]. Presently, BPC 157 certainly does not fulfill the general standard neurotransmitter criteria, as defined in classic terms [12,13,14]. The BPC 157 research did not provide direct conclusive evidence that the chemical is produced within the neuron or is present in it as a precursor molecule, released eliciting a response on the receptor on the target cells on neurons and being removed from the site of action once its signaling role is complete. Likewise, the whole peptide BPC from human gastric juice and BPC 157 as the active terminal fragment presentation [15] apparently departs from the standard neuropeptide characteristics, identification from the biological activities, identification from the receptor, identification from biochemical characteristics, identification using genomic or peptidomic approaches, carefully reviewed [34,35]. 

On the other hand, there can be favoring the BPC 157/neurotransmitter relations. This goes with its ample range of beneficial effects, obtained within the same dose range [1,2,3,4,5,6,7,8,9,10,11,15,27,28,29,30,33,109,124,125], as the most valuable proof of the neurotransmitter requires that the application of the chemical directly to the target cells should produce the same response observed when the chemical is naturally released from the neurons [12,13,14]. Thus, given the particularities of the observed BPC 157 therapy effect [1,2,3,4,5,6,7,8,9,10,11,15,27,28,29,30,33,109,124,125], this final point, the equation between the exogenous effects and endogenous presence to verify endogenous presence and significance, can be a suggestive argument for the possible BPC 157/neurotransmitter relation.

**Figure 1:**
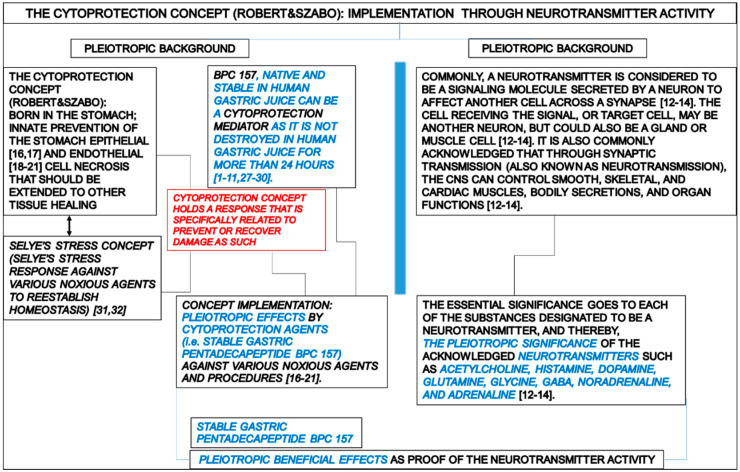
The cytoprotection concept (Robert and Szabo): implementation via neurotransmitter activity. The stable gastric pentadecapeptide BPC 157 pleiotropic beneficial activity and its possible relations with neurotransmitter activity based on the general significance of Robert’s and Szabo’s stress concept and Selye’s stress concept leading to the pleiotropic effects by cytoprotection agent application (BPC 157 acts as a cytoprotection mediator, and thereby pleiotropic significance), and pleiotropic significance of acknowledged neurotransmitters (i.e., acetylcholine, histamine, dopamine, glutamine, glycine, GABA, noradrenaline, and adrenaline). For the stable gastric pentadecapeptide BPC 157, pleiotropic beneficial effects can be used as proof of the neurotransmitter activity [1,2,3,4,5,6,7,8,9,10,11,12,13,14,16,17,18,19,20,21,27,28,29,30,31,32]. All these points may be supported by its special interaction with various molecular pathways [8,11,75,115,116,117,118,119,120,121,122,123], especially with the nitric oxide (NO)-system [121,122,123,124,125,126,127,128,129,130] as a whole. Note, in general, since the very beginning, the concept of cytoprotection did not consider the neurotransmitter activity as the basis for the cytoprotective effects of the implemented agents (i.e., prostaglandins [16,17], sulfhydryl [141], somatostatin [142], dopamine [143]).

Therefore, the conceptual evidence can be summarized as follows (Figure 1).

### 1.1. Dopamine

**Figure 2:**
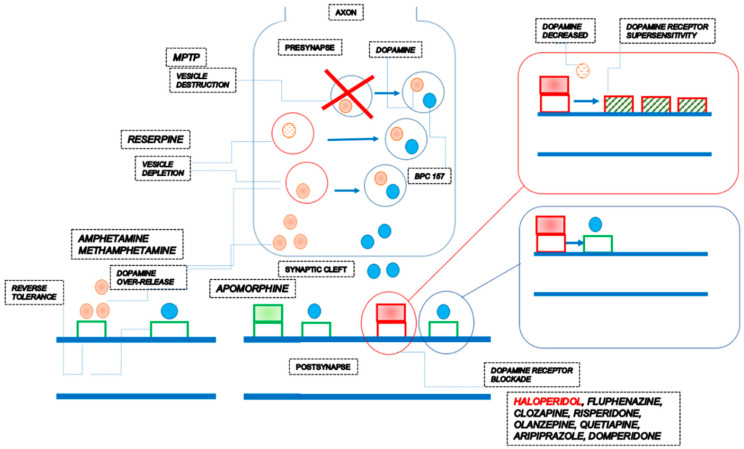
The stable gastric pentadecapeptide BPC 157 pleiotropic beneficial activity and its possible relations with dopamine neurotransmitter activity assuming BPC 157 own presence in vesicles. There is an effect of BPC 157 on dopamine as a whole, maintained vesicle integrity and function (i.e., counteraction against reserpine- or amphetamine—and MPTP-effect) [69,70,152], prevented amphetamine-disturbances, reversed already advanced disturbances, and counteracted amphetamine “reverse tolerance” even after a very long period [70,153], and even substitute the loss of dopamine neurons (MPTP) (note, BPC 157 counteracted tremor, rigor, akinesia, and also MPTP-mortality) [69], and competing successfully for sites at dopamine receptors (i.e., counteracting the adverse effects of both dopamine agonists [70,152,153] and dopamine antagonists [68,70,152]). A final proof by BPC 157 therapy is the counteraction of the dopamine receptor supersensitivity as a general phenomenon, given that counteraction confirms the resolution of the otherwise determining issue of decreased dopamine synthesis [152,164].

In principle, it is commonly acknowledged that altering neurotransmitter activity by drugs influences behavior while targeting the neurotransmitter of major systems affects the whole system. Thus, the complexity of the actions of some drugs can reveal their connection with the role of neurotransmitters [12,13,14]. There, in dopamine/BPC 157 relations, BPC 157 can have a special role in providing a wide range of influence on various, even opposite, activities [1], likely suggestive of its neurotransmitter role on its own. Note that BPC 157 by itself does not induce any behavioral change [68] and was claimed to have a modulatory role, as mentioned [1]. BPC 157 therapy can counteract the consequences of dopamine neurons destruction in the substantia nigra (neurotoxin MPTP) [69], dopamine vesicle depletion (reserpine) [69], dopamine receptors blockade (neuroleptics) [68,70], dopamine over-release and re-uptake inhibition (amphetamine; methamphetamine) [70,152,153], dopamine receptor agonization (apomorphine) [70], dopamine receptor supersensitivity (haloperidol) [152] and reverse tolerance (amphetamine) [153]. In all of these distinctive disturbances, BPC 157 therapy is effective within the same dose range [68,69,70,152,153] (Table 1, Figure 2).

Noteworthy, each of these models has a particular target, and they are properly oriented as suited models, i.e., Parkinson’s disease (MPTP, reserpine) [69], catalepsy (neuroleptics) [68,70], and schizophrenia (positive symptoms) (amphetamine) [70,152]. Further, these models received common acknowledgment; they can speak for themselves, and we can follow each of the destructive specific chains of events toward the complex common processes that remain to be further defined and resolved. Given the well-defined background of each of the given agents and all of them as a general dopamine issue [68,69,70,152,153], we can suggest that the counteraction by BPC 157 therapy can be injury-related, to compete directly or indirectly with the processes mentioned below as a whole in a particular highly orchestrated way.

The size of the neurotransmitter pools decreases with reserpine. Days to weeks to replenish the depleted vesicular monoamine transporters (VMATs), monoamine oxidase (MAO), as well as catechol-O-methyltransferase (COMT), metabolize unprotected neurotransmitters, given the post-synaptic cell are consequently never excited [154,155].

Likewise, MPTP is metabolized into the toxic cation 1-methyl-4-phenylperydinium (MPP^+^) [156] by the enzyme MAO-B of glial cells, specifically astrocytes, provides that high-affinity for MPP^+^ affects dopamine transporter (DAT). Consequently, dopamine neurons exhibit dopamine reuptake and are unable to transport dopamine back into the cells, and loss of dopamine neurons occurs [157].

Shortage or excess of dopamine can prevent proper function and signaling of these receptors, leading to disease states [158]. Amphetamine increases the concentration of dopamine in the synaptic cleft by entering the presynaptic neuron by diffusing across the neuronal membrane directly or through DAT, competitive reuptake inhibition at the transporter, and finally, the reversal of dopamine transport through DAT (i.e., dopamine efflux) [159,160,161]. Apomorphine, as a non-selective agonist, activates both D2-like and, to a much lesser extent, D1-like receptors [162].

Antipsychotic drugs have a similar blocking effect on D2 receptors [163]. A general phenomenon after a single injection of neuroleptics is the development of DA receptor supersensitivity, and the degree and duration of this supersensitivity are related to the degree and duration of the preceding receptor blockade [164]. In addition, the time of shift from receptor blockade to supersensitivity coincides rather closely with the shift from increased to decreased DA synthesis [164].

**Table 1:** A restorative dimension of the BPC 157 therapy can react with the dopamine system depending on the condition [68,69,70,152,153].

Effect	Specification	Ref.
BPC 157 therapy can counteract the consequences of dopamine neurons destruction in the substantia nigra (neurotoxin MPTP)	BPC 157 counteracted tremor, rigor, akinesia, and MPTP mortality. BPC 157 counteracted MPTP-induced gastric lesions.	[69]
BPC 157 therapy can counteract the consequences of dopamine vesicle depletion (reserpine)	BPC 157 counteracted tremors, rigor, akinesia, and hypothermia. BPC 157 counteracted reserpine-induced gastric lesions.	[69]
BPC 157 therapy can counteract the consequences of dopamine receptor blockade (neuroleptics)	BPC 157 therapy counteracted catalepsy and somatosensory disorientation induced by dopamine antagonists [68,70]. BPC 157 therapy counteracted prolonged QTc intervals [89], gastric lesions, and lower esophageal sphincter and pyloric sphincter dysfunction induced by dopamine receptor antagonists [68,92,165], as well as gastric lesions induced by combined application of dopamine receptor antagonist and reserpine [166]. A common effect is a counteraction of occlusion/occlusion-like syndromes, peripherally and centrally, induced by haloperidol, fluphenazine, clozapine, risperidone, olanzapine, quetiapine, aripiprazole and domperidone [54]. BPC 157 therapy (activation of the collateral pathways, i.e., azygos vein direct flow delivery) instantly occurred.	[68,70]
BPC 157 can prevent and reverse amphetamine disturbances (and methamphetamine), acutely and chronically, schizophrenia positive symptoms-like models.	BPC 157 can prevent amphetamine disturbances, reverse already advanced disturbances, and counteract amphetamine “reverse tolerance” even after a very long period (i.e., 46 days) [70,153]. A common effect is a counteraction of occlusion/occlusion-like syndromes, peripherally and centrally, induced by amphetamine [54]. BPC 157 therapy (activation of the collateral pathways, i.e., azygos vein direct flow delivery) instantly occurred.	[70,153]
BPC 157 can reverse apomorphine motor disturbances, schizophrenia-positive symptoms-like models	BPC 157 can reverse continuous oral stereotypy (licking, gnawing) in apomorphine rats.	[70]
The counteraction of the dopamine receptor supersensitivity (and thereby counteraction of amphetamine over-activity in haloperidol pretreated mice, simultaneous counteraction of both haloperidol and amphetamine effects.	There is a prominent effect of BPC 157 on increased amphetamine-climbing behavior in mice pretreated with dopamine antagonists haloperidol (5 mg/kg ip) and subsequently treated with amphetamine (20 mg/kg ip challenge at 1, 2, 4, and 10 days after haloperidol pretreatment. An almost complete reversal occurred when BPC 157 was coadministered with haloperidol.	[152]
In conclusion, there is a restorative dimension of the BPC 157 therapy, given that it can react with the dopamine system depending on the condition [68,69,70,153].	The restorative dimension of the BPC 157 therapy, and thereby BPC 157 activity over the dopamine system, as a likely neurotransmitter of its own, can be based on the following consistent evidence providing a wide range of influence on various, even opposite, activities [1]. BPC 157 therapy can counteract the consequences of dopamine neurons destruction in the substantia nigra (neurotoxin MPTP) [69], dopamine vesicle depletion (reserpine) [69], dopamine receptors blockade (neuroleptics) [68,70], dopamine over-release and re-uptake inhibition (amphetamine; methamphetamine) [70,152,153], dopamine receptor agonization (apomorphine) [70], dopamine receptor supersensitivity (haloperidol) [152] and reverse tolerance (amphetamine) [153].	

Thus, although altering many of these points was not specifically demonstrated, the firm effect of BPC 157 therapy can be a valid judgment based on the consistent counteraction of the behavioral effects of all of the mentioned dopamine agents and evidence that the exogenous agent’s administration should likely mimic its endogenous significance. Likely, BPC 157 should have its own presence in vesicles. Namely, it can maintain vesicle integrity and function (i.e., VMATs, DAT) (i.e., counteraction against reserpine- or amphetamine—and MPTP-effect) [69,70,152]. Note, for BPC 157, a persistent effect is mandatory, as it can prevent amphetamine disturbances, reverse already advanced disturbances, and counteract amphetamine “reverse tolerance” even after a very long period (i.e., 46 days) [70,153] and even substitute the loss of dopamine neurons (MPTP) (note, BPC 157 counteracted tremor, rigor, akinesia, and also MPTP-mortality) [69]. When released (e.g., with vesicle depletion by reserpine or amphetamine) [69,70,152] it can substitute disturbed dopamine (i.e., shortage (reserpine) [69] or excess (amphetamine) [70]) as competing successfully for sites at dopamine receptors (i.e., counteracting the adverse effects of both dopamine agonists [70,152,153] and dopamine antagonists [68,70,152]). A final proof by BPC 157 therapy (not achievable by dopamine agents) is the counteraction of the dopamine receptor supersensitivity (and thereby counteraction of amphetamine over-activity in haloperidol pretreated mice) as a general phenomenon given that counteraction confirms the resolution of the otherwise determining issue of decreased dopamine synthesis [152,164]. This can be performed with the assistance of NO-system, given that in apomorphine or amphetamine (acute application) and methamphetamine (chronic application) rats, BPC 157 therapy counteracts the effects of NO-agents’ application [70]. Thus, given BPC 157 activities as such, or BPC 157 activities as neurotransmitters, a restorative dimension to a damaged or dysfunctional dopaminergic system can be a strong argument for its general significance [68,69,70,152,153].

This can be further extended. Likely, strong counteraction of amphetamine by BPC 157 therapy can signify the counteraction of other amphetamine effects (the increased release of dopamine in the nucleus accumbens and striatum), such as many changes in brain morphology and physiology. These can be the upregulation of alpha-amino-3-hydroxy-5-methyl-4-isoxazolepropionic acid (AMPA) receptors, altered NMDA receptor expression, altered dendritic morphology [167,168], and altered functioning of cholinergic (ACh-ergic) basal neurons, projecting to the cortex [169] by repeated exposure to amphetamine (amphetamine-induced sensitization). In addition, it has been suggested that the deficits in attentional function induced by amphetamine may be connected to the dysregulation of ACh-ergic activity [170]. Further, given the findings in other BPC 157 studies [1,3,4], it can be that on a molecular level, BPC 157 can interfere with increased tumor necrosis factor-alpha (TNF-alpha) and malondialdehyde (MDA) levels [8,11], suggesting ongoing increased inflammatory and oxidative processes that can be induced by dopaminergic enhancers, such as amphetamine [171].

Further, given that via synaptic transmission (also known as neurotransmission), the CNS can control smooth, skeletal, and cardiac muscles, bodily secretions, and organ functions [12,13,14], particular BPC 157 therapy effects should be emphasized in no-behavioral studies. These were prolonged QTc intervals [89], gastric lesions, and lower esophageal sphincter and pyloric sphincter dysfunction induced by dopamine receptor antagonists [68,92,165,172], as well as gastric lesions induced by combined application of dopamine receptor antagonist and reserpine [166]. These were all counteracted by BPC 157 therapy.

Finally, quite recently, typical neuroleptics, haloperidol and fluphenazine, atypical neuroleptics, clozapine, risperidone, olanzapine, quetiapine, and aripiprazole, antiemetic peripherally acting, domperidone, and dopamine indirect agonist amphetamine as a common effect have occlusion/occlusion-like syndrome [54]. Likewise, as a common effect in counteraction of occlusion/occlusion-like syndromes, peripherally and centrally, there is a BPC 157 therapy (activation of the collateral pathways, i.e., azygos vein direct flow delivery instantly occurred) [40,41,42,43,47,48,49,50,51,52,53,54,55,56], in particular with occlusion/occlusion-like syndrome induced by either of those dopamine agents [54]. Moreover, after various neuroleptics, typical and atypical, peripherally acting domperidone, and amphetamine, BPC 157 therapy attenuated/eliminated as a general effect vascular and multiorgan failure, major vessel failure, thrombosis, advanced Virchow triad circumstances, and blood pressure disturbances (intracranial (superior sagittal sinus), caval and portal hypertension, and aortal hypotension) both peripherally and centrally [54]. These effects (i.e., rapidly reestablished reorganized blood flow) attenuated/eliminated the lesions in the brain (intracerebral and intraventricular hemorrhage), heart (congestion, myocardial infarction, severe arrhythmias), lung (hemorrhage), and congestion and lesions in the liver, kidney, and gastrointestinal tract [54].

### 1.2. Serotonin

In terms of serotonin and its possible neurotransmitter role on its own, BPC 157 can have a special role given a wide range of activities. The arguments are its particular combination, such as antidepressant effect (Porsolt’s test, open field) [65] and region-specific influences on brain serotonin synthesis in rats given a particular increase in the substantia nigra [67] vs. counteraction of the serotonin syndrome as a whole [66]. Further, there is a reduction in enteric serotonin concentration, attenuated intestinal motility, increased survival of cultured enteric neurons, the proliferation of cultured EGCs [119], and a particular effect on maintaining platelets function [128,129,130] (Table 2).

Of note, serotonin has a particular multifacetedness given all functions involved (i.e., mood, cognition, reward, learning, memory, and numerous physiological processes). Illustratively, it is produced in the central nervous system (i.e., brainstem’s raphe nuclei) (1–2% of the serotonin in the human body), skin (i.e., Merkel cells), lung (pulmonary neuroendocrine cells), gastrointestinal tract (90% of the serotonin in the human body) (i.e., enterochromaffin cells), and rest of 8% is stored in platelets [166].

The regional serotonin synthesis (using alpha-[14C]methyl-L-tryptophan (α-MTrp) autoradiographic measurements) in the brain following peripheral (intraperitoneal) BPC 157 administration is subjected to particular, likely indicative changes, timely and brain areas specific [67]. There was an overall decrease in brain serotonin synthesis following acute treatment, which provided an overall increase in synthesis in chronically treated rats [67]. In acute treatment, a significant decrease in the globus pallidus, dorsal and ventral hippocampus, dorsal thalamus, lateral geniculate body, and hypothalamus [67]. Contrarily, the synthesis significantly increased in the medial anterior olfactory nucleus and substantia nigra reticulate. In chronic treatment, a significant decrease observed in the dorsal raphe is along with increases in the substantia nigra, the lateral caudate, and the accumbens nucleus. This can likely point to a particular serotonergic response that is timely related to BPC 157 applications. The substantia nigra’s (compacta and reticulata) structure (given serotonin synthesis significantly increased following both acute and chronic treatments) occurred as a particular point of pentadecapeptide BPC 157 [67]. This can construct a particular BPC 157 pathway along with that of serotonin and dopamine, given the dense presence of dopaminergic neurons and dense projections of the serotonergic systems [175]. This is also the above-mentioned evidence that BPC 157 therapy within the same dose range counteracted the consequences of dopamine disturbances. As mentioned, counteracted were neurons destruction in the substantia nigra (MPTP) [69], dopamine vesicle depletion (reserpine) [69], dopamine receptors blockade (neuroleptics) [68,70], dopamine over-release and re-uptake inhibition (amphetamine; methamphetamine), dopamine receptor agonization (apomorphine) [70] and dopamine receptor supersensitivity (haloperidol) [152] and reverse tolerance (amphetamine) [153]. Note that the metabolic pathway of α-MTrp follows [176] the biosynthetic route of serotonin from tryptophan (Trp) [177].

Furthermore, the value of such effects of BPC 157 reported [67] (which do not resemble the results obtained with any other serotonergic drug using this method [178,179,180,181]) is fully supported by the counteracting potential of BPC 157 therapy on serotonin syndrome [66]. The development of serotonin syndrome is one of the most serious side effects of antidepressant therapies [182]. Commonly, the serotonin syndrome results from an excess of synaptic serotonin following ingestion of drugs that enhance serotonergic transmission either by increasing serotonin synthesis or release or by inhibiting serotonin uptake or degradation [182]. Several classes of antidepressant drugs have been implicated in the serotonin syndrome, including serotonin reuptake inhibitors, tricyclic antidepressants, and monoamine oxidase inhibitors) [183,184]. The risk of precipitating the serotonin syndrome is increased if serotonergic drugs with different mechanisms of action are ingested, even weeks apart [182,183,184]. The ability of tricyclic antidepressants and serotonin reuptake inhibitors to elicit the serotonin syndrome correlates with the particular drug’s affinity for the 5-HT transporter (5-HTT) [183]. Additional drugs that may be causative of the serotonin syndrome include the amino acid precursors of serotonin, tryptophan, and 5-hydroxy-l-tryptophan (5-HTP) [182]. Increased availability of serotonin precursors augments the amount of available serotonin, which might lead to the development of the serotonin syndrome [182] and, thereby, the involvement of both 5-HT1A and 5-HT2A receptors.

First, BPC 157 therapy counteracted serotonin syndrome initiation (i.e., counteracted pargyline effect) [66]. Then, in particular, BPC 157 counteracted the full serotonin syndrome crisis (attenuated the adverse effect of the subsequent L-tryptophan application) [66]. This effect may have been a particular effect, as BPC 157 counteracted each part of the serotonin syndrome presentations, and then, BPC 157 therapy might fully counteract serotonin syndrome [66] as a whole. Namely, the irreversible monoamine oxidase (MAO B) inhibition (i.e., pargyline) and subsequent serotonin substrate (L-tryptophan as a serotonin precursor) induced fore paw treading, hind limbs abduction, wet dog shake, and hypothermia followed by hyperthermia in rats, which commonly occur in serotonin syndrome [66]. Both temperature and behavioral changes in all these experiments were counteracted by gastric pentadecapeptide BPC 157. Thus, given the effect on serotonin brain synthesis and counteraction of the serotonin syndrome, it appears that BPC 157, in a particular way, can control the whole chain of serotonin events. Of note, this can also go to the above-mentioned counteracted effects of amphetamine. Amphetamine exerts analogous, yet less pronounced, effects on serotonin via VMAT2 as on dopamine and norepinephrine [160,185]. Finally, in classic antidepressant assays, BPC 157 therapy (Porsolt’s test, chronic stress, reduced duration of immobility) overwhelmed the effect of imipramine [65].

Further support for BPC 157/serotonin relation is the inhibited release of enteric serotonin and inhibited intestinal motility, the increased survival rate of cultured enteric neurons, and the increased proliferation of cultured enteric glial cells (EGCs) by BPC 157 application [119]. These findings were carried out to emphasize the cytoprotection potential of BPC 157 therapy [119] and can together provide an additional particular hallmark for serotonin balance by BPC 157 application [119]. Likewise, BPC 157 can maintain platelet function in a particular way [128,129,130]. Note that studies have suggested that EGCs would exert essential roles in supporting the survival and functions of the ENS neurons [186]. Notably, recent evidence reveals that EGCs could possess multiple immune functions and thereby may participate in the immune homeostasis of the gut [186]. In addition, for maintained platelet function, peripherally and centrally as a particular vascular and multiorgan failure occlusion/occlusion-like syndrome, above described, was clearly responsive to the BPC 157 therapy application [40,41,42,43,47,48,49,50,51,52,53,54,55,56], given eliminated/annihilated hemorrhage (i.e., brain, lung) and thrombosis, in particular of consideration of pulmonary embolism [87] and evidently, reversed already advanced Virchow triad circumstances [40,41,42,43,47,48,49,50,51,52,53,54,55,56]. In addition, there was the specifically maintained thrombocytes function (i.e., the opposed L-NAME-pro-thrombotic effect, opposed L-arginine-anti-thrombotic effect) [128], given the coagulation pathways not affected as also demonstrated in aggregometry and thromboelastometry studies [128,129,130], and counteracted prolonged bleeding following anticoagulants (heparin, warfarin) and anti-platelet agents (aspirin, clopidogrel, cilostazol) [126,127,128], organ perforation [49,173,174] or amputation of tail or foot [128,129,130].

Thus, given these findings together, it can be an argument analogy between the BPC 157 presentation and the presentation of serotonin, which is most commonly acknowledged [166]. Native and stable in human gastric juice, BPC 157 can be released into the circulation and sent to distant organs by complex biological processes to regulate physiology and behavior [1,2,3,4,5,6,7,8,9,10,11,15,27,28,29,30,33,37]. The most commonly acknowledged serotonin presentation can be from the enterochromaffin cells out of tissues into the blood, secreted luminally and basolaterally increased serotonin uptake by circulating platelets and activation after stimulation, toward the increased stimulation of myenteric and gastrointestinal motility [187]. This can acknowledge that the essential remainder (1–2%) synthesized in serotonergic neurons of the CNS and various functions [166] can have an analogous presentation of the innate BPC 157 systems and functions [1,2,3,4,5,6,7,8,9,10,11,15,27,28,29,30,33,37]. Therefore, this can be a restorative dimension of the BPC 157 therapy, which is able to react depending on the condition [65,66,67].

### 1.3. Glutamate

*N*-methyl-D aspartate-type glutamate receptor (NMDAR) uncompetitive antagonists, such as phencyclidine and ketamine (phencyclidine derivative), transiently induce symptoms and cognitive deficits characteristic of schizophrenia in humans [188,189,190,191,192,193]. This observation substantiated the glutamate hypothesis and glutaminergic dysfunction as a mechanism underlying both positive and negative symptoms, as well as cognitive dysfunction, in schizophrenia [194]. Thereby, ketamine as the model of schizophrenia in rats [193] (as acute ketamine administration was associated with schizophrenia-like or psychotomimetic symptoms with large effect sizes and an increase in positive and negative symptoms [195,196]) might escape from the extraordinary complexity of extrapolation from animal models of mental disorders in general [197,198,199].

BPC 157/glutamate relation goes with the evidence that BPC 157 counteracted negative-like schizophrenia symptoms, ketamine-cognition dysfunction, social withdrawal, and anhedonia and exerted additional anxiolytic effects in rats [59] (Table 3).

These findings should be overseen with glutamate as the major excitatory neurotransmitter in the central nervous system (i.e., cognitive functions such as learning and memory in the brain [200], the precursor of major inhibitory neurotransmitter GABA) but excitotoxic neuronal death as a direct result of high concentrations [201], and tight physiologic range that should be maintained [59]. Thereby, in general terms, the counteracted all negative-like symptoms of schizophrenia in ketamine-rats, the counteracted ketamine-cognition dysfunction, social withdrawal, and anhedonia, and exerted additional anxiolytic effect by BPC 157 therapy should be some suited control [59]. This should be a control of the release of glutamate in the synaptic cleft from presynaptic terminals to stimulate post-synaptic neurons (i.e., the NMDA receptors) and re-uptake from the cleft by the excitatory amino acid transporters (EAAT) into astrocytes, and glutamate/glutamine cycle within astrocytes [59]. This should be a particular effect on ketamine functions given the counteracted ketamine-cognition dysfunction, counteracted social withdrawal, and counteracted anhedonia versus exerted additional anxiolytic effect [59]. Note that the antidepressant and anxiolytic effect of ketamine was largely reviewed [202,203]. Possibly, BPC 157 can collaborate with NMDA receptor inhibitors to restore hippocampal function in the stressed brain by reducing NMDA receptor activity in animal models [204]. Refractory anxiety may cause glutamate receptor damage in the hippocampus and neuronal atrophy in the prefrontal cortex (PFC), which can result in mood disturbances and cognitive deficits [205]. Further, there is partial ketamine agonism for the high-affinity state of the D2 dopamine receptor and ketamine’s weaker affinity for serotonin 5-HT2A receptors [206]. This can be seen along with the above-mentioned BPC 157 on dopamine- and serotonin-system, and thereby, full effectiveness (positive and negative symptoms of schizophrenia [59,70], antidepressant effect [65,66,67], serotonin syndrome counteraction [67]). Thereby, BPC 157 effects on counteraction of ketamine-cognition dysfunction, social withdrawal, and anhedonia, and exerted additional anxiolytic effect, in addition to well-suited models (i.e., cognitive dysfunction (novel object recognition test)) [207,208,209], social withdrawal [210,211], anhedonia (sucrose test) [212,213], anxiogenic effect (open field test) [214,215], can have a particular background. Further, BPC 157 counteracted the effect of MK-801, a non-competitive antagonist of the NMDA receptor application [70], locomotion, stereotyped sniffing, and ataxia as a sign of positive-like symptoms of schizophrenia [70]. MK-801 (dizocilpine) has a greater inhibitory potency than ketamine and phencyclidine [216].

The significance of such counteraction is further established by the additional application of NO-agents [59]. NO-system organized therapy (i.e., simultaneously implied NO-system blockade (L-NAME) vs. NO-system over-stimulation (L-arginine) vs. NO-system immobilization (L-NAME+L-arginine)) reveals that each of „negative symptoms“ differs from each other given their different responsibility to L-NAME and L-arginine given alone or together. There is a considerable extent of the noted NO-differences, and the distinctive brain areas supposed to be affected by specific ketamine effects (given overall glutamate brain presence vs. dopamine striatal brain regions (positive symptoms of schizophrenia as an excess of dopaminergic neurotransmission)), and prefrontal brain regions (negative symptoms and cognitive deficits linked to dopaminergic hypofunction) [195,217]. Further, there is a distinctive ketamine dosage range. All are counteracted using the same dosage range of BPC 157, and together, they can indicate the widespread presence of possible BPC 157 pathways [59].

Interestingly, given that BPC 157 by itself does not induce any behavioral change as mentioned, we identified a similar overexpression of the genes in the healthy rats treated with the ketamine (Nos1, Nos2, Plcg1, Prkcg, Ptgs2, and Ptk2) and in the BPC 157 (Nos1, Nos2, Plcg1, Prkcg, and Ptk2). Thus, we revealed a considerable overlapping of gene overexpression [59]. Thus, the evidenced effect on the given gene expression in the brain tissue of the BPC 157 therapy applied immediately after ketamine provided particular insight. There was Nos1 (decreased expression), Nos2 (increased expression), Plcg1 (decreased expression), Prkcg (increased, and then decreased expression), Ptgs2 (increased expression), and no effect on Nos3 and Ptk2. Likely, this may be a timely specific BPC 157 effect on ketamine-specific brain targets [59]. Conceivably, this may indicate the way BPC 157, given peripherally, may specifically interfere with the ketamine-induced effects, likely on the specific brain targets, regardless of the possible limitation of results only reflecting mRNA levels, which may not correlate with protein levels [38]. Thus, this might be a direct effect of BPC 157–ketamine.

### 1.4. GABA

BPC 157/GABA relation goes with the same supportive evidence as in the dopamine, serotonin, and glutamate studies (see Chapters Dopamine, Serotonin, Glutamate), a restorative dimension, as mentioned before [1]. BPC 157 has a particular anxiolytic effect on its own (light/dark, shock probe/burying [57], ketamine [59]). Further, in diazepam tolerance/withdrawal and physical dependence/withdrawal studies [58], BPC 157 coadministration in chronically treated diazepam mice counteracts diazepam tolerance and withdrawal, postpones physical dependence, and prolongs residual diazepam anticonvulsive activity [58]. BPC 157 therapy also, on its own, counteracts isoniazid (GABA synthesis inhibitor)-, picrotoxin (non-completive channel blocker for GABAA receptors (GABAARs) chloride channels)- [58], and bicuculline (completive antagonists of GABAARs)-convulsions [15]. Finally, BPC 157 therapy counteracts the anesthetic effect of thiopental in rats [218], a prototype member of the barbiturate class of drugs (Table 4).

Thus, BPC 157 therapy can interfere in a controlling particular way with diazepam tolerance as a mechanism operative in neuronal circuit adaptation to the extreme amplification of GABA-gated Cl^−^ current intensities [219]. Commonly, tolerance results in a desensitization of GABA receptors. There is also an increased sensitization of the excitatory neurotransmitter system, such as NMDA glutamate receptors, the reduction in the number of GABA receptors (downregulation), the shift of benzodiazepine receptors to an inverse agonist state, and compensatory changes in other components of the macromolecular GABA/benzodiazepine/chloride ionophore complex (for review see, i.e., [219,220,221,222,223,224,225]). This can be important given GABAARs, as members of the Cys-loop family of pentameric transmembrane ligand-gated ion channels, expressed in parts of the nervous system that process higher-order brain functions and have influences on regulating synaptic transmission and integration of synaptic signals central to memory, awareness, and consciousness [226].

Further, such particular BPC 157 restoring GABA capacity in all particular points [1] is further supported by a consistent counteraction of convulsion induced by isoniazid, picrotoxin, and bicuculline [15,58]. This can be indicative by providing all distinctive points affected by convulsants [227]; consequently, all affected by BPC 157 therapy were given counteracted convulsions. Isoniazid has a depleting brain level of GABA through inhibition of pyridoxal-5-phosphate-dependent glutamic acid decarboxylase (GAD) [226,228]. Picrotoxin acts as a competitive antagonist of GABAARs, acting on synapses as a non-competitive channel blocker for GABAARs chloride channels, specifically the GABA-activated chloride ionophore [229]. Bicuculline is a competitive antagonist of GABAARs primarily on the ionotropic GABAARs, ligand-gated ion channels, and the passing of chloride ions across the cell membrane [230].

Finally, BPC 157 has a considerable potential to counteract thiopental anesthesia (i.e., BPC 157 in doses of 10 ng/kg and 10 µg/kg, respectively, caused significant counteraction of loss of righting reflex produced by thiopental with a parallel shift of the dose–response curve for thiopental to the right) [218]. Thereby, counteraction goes to GABAAR currents increased by barbiturates (and other general anesthetics) [218]. Note that this can even have an extended effect on BPC 157 therapy and BPC 157 itself. Illustratively, BPC 157 therapy also counteracts the effect of L-NAME, which increases the thiopental loss of the righting reflex seven times. Furthermore, while GABAAR currents are increased by barbiturates (and other general anesthetics), barbiturates, as relatively non-selective compounds, bind to an entire superfamily of ligand-gated ion channels and can block ligand-gated ion channels predominantly permeable for cationic ions, the neuronal nicotinic acetylcholine receptor (nAChR), the 5-HT3 receptor, the glycine receptor, and others [231,232]. There is a cross-tolerance between alcohol, benzodiazepines, barbiturates, nonbenzodiazepine drugs, and corticosteroids, which all act by enhancing the GABAA receptor’s function via modulating the chloride ion channel function of the GABA_A_ receptor [233,234,235,236]. 

Alcohol has a very similar mechanism of tolerance and withdrawal to benzodiazepines, involving the GABAA, NMDA, and AMPA receptors [237], and thereby, the extent of this counteraction should be indicative of the observed effects [63,64]. Alcohol binds strongly to GABA receptors, activating the inhibitory cascade, which results in sedation, cognitive dysfunction, and decreased coordination. With chronic alcohol use, the number of GABA receptors is increased, requiring more and more alcohol to create the same level of inhibition. This is a phenomenon known as tolerance. This tolerance partly explains the alertness of chronic alcohol users at blood alcohol levels that, in others, would cause coma or death [238,239,240,241]. Indicative is the counteraction of both acute and chronic alcohol effects as a highlight of the BPC 157 particular potential consistently evidenced in mice that were either acutely intoxicated or physically dependent on alcohol [63,64]. BPC 157 intraperitoneally or intragastrically strongly prevented and reversed the effects of acute intoxication (i.e., quickly produced and sustained anesthesia, hypothermia, increased ethanol blood values, 25% fatality, 90 min assessment period) when given before or after ethanol, and none of the mice died. When given after abrupt cessation of chronic ethanol (at 0 or 3 h withdrawal time), it attenuated withdrawal and handling induced withdrawal seizures [63,64]. Furthermore, in addition to counteracting acute and chronic alcohol intoxication, the therapy includes both central and peripheral lesions, mucosal and endothelial lesions, liver injuries, and portal hypertension counteraction [242,243].

Finally, following acute absolute alcohol intragastric administration, BPC 157 therapy attenuated/eliminated the alcohol-occlusion/occlusion-like syndrome as a whole, major vascular and multiorgan failure [56], as described above [40,41,42,43,47,48,49,50,51,52,53,54,55,56]. Intracranial, portal, and caval hypertension and aortal hypotension, lesions and hemorrhage in the brain, heart, lung, liver, and kidney, and thrombosis peripherally and centrally were all counteracted along with counteraction of the prime major stomach alcohol lesion [56]. The therapy effect was ascribed to the counteraction of the congestion of major vessels, and particularly to activation of the rescuing collaterals, i.e., azygos vein direct blood flow delivery. BPC 157 therapy effectiveness illustrates that brain swelling instantly decreases [56]. A fall of intracranial hypertension occurs immediately upon BPC 157 therapy [56]. These effects were also shown to be NO-system dependent [64,124,125].

### 1.5. Acetylcholine

BPC 157/acetylcholine relation principle can be a particular way, noted in particular with BPC 157 counteraction of the succinylcholine [81], rocuronium (report in preparation), pilocarpine [244] and atropine [86] effects. BPC 157, given intragastrically or intraperitoneally in the same dose range, can considerably antagonize the course of succinylcholine and neuromuscular block as a whole [81]. Thereby, it can counteract agitation before muscle disability, numerous twitches before complete loss of muscle tone, motionless prostration, and, subsequently, a painful reaction (violent screaming upon light touch) [81]. Otherwise, succinylcholine, as not hydrolyzed by acetylcholinesterase, has a longer duration of effect than acetylcholine, and by maintaining the membrane potential above the threshold, muscle cells could not be repolarized. When acetylcholine binds to an already depolarized receptor, it cannot cause further depolarization [245,246,247]. 

Thus, it can be, given the antagonizing potential of BPC 157 therapy, that BPC 157 can antagonize all of these effects or substitute acetylcholine (dys)function [81] (Table 5).

In addition, to support such a particular effect, BPC 157 dose-dependently counteracted the effect of rocuronium (report in preparation) (i.e., nondepolarizing (competitive) neuromuscular blockade, antagonizing nicotinic acetylcholine receptors directly, preventing binding of endogenously released acetylcholine and subsequent muscle cell depolarization). Additionally, BPC 157 counteracted the effect of muscarinic receptor agonist pilocarpine, miosis (and subsequent mydriasis in rats due to muscle disability), hypersalivation, and convulsions [247]. Contrarily, BPC 157 counteracted prototypical muscarinic receptor antagonist atropine and mydriasis in rats and guinea pigs [86]. An equal counteracting effect following local, intragastric, and intraperitoneal application [86] is consistent with an overall effect on acetylcholine system function. These effects appear to be NO-system related [86]. Note that, unlike humans, mydriasis occurred with pilocarpine in mice and rats [248,249,250].

### 1.6. Adrenaline/Noradrenaline

As a common effect including counteraction of the sotalol, class II and class III antiarrhythmic and beta-blocker [52], and counteraction of the isoprenaline, a prototype of beta-agonists [53], occurred counteraction of occlusion/occlusion-like syndromes, peripherally and centrally, and there is a BPC 157 therapy (activation of the collateral pathways, i.e., azygos vein direct flow delivery instantly occurred) [40,41,42,43,47,48,49,50,51,52,53,54,55,56]. After either sotalol [52] or isoprenaline [53], BPC 157 therapy attenuated/eliminated occlusion/occlusion-like syndrome as a general effect vascular and multiorgan failure, major vessel failure, thrombosis, advanced Virchow triad circumstances, and blood pressure disturbances (intracranial (superior sagittal sinus), caval and portal hypertension, and aortal hypotension) both peripherally and centrally [53]. These effects (i.e., rapidly reestablished reorganized blood flow) attenuated/eliminated the lesions in the brain (intracerebral and intraventricular hemorrhage), heart (congestion, myocardial infarction, severe arrhythmias), lung (hemorrhage), and congestion in the liver, kidney, and gastrointestinal tract [52,53]. In rats challenged with sotalol, BPC 157 therapy was 10 µg, 10 ng/kg given intragastrically at 5 min or 90 min sotalol-time [52]. In isoprenaline rats, BPC 157 (10 ng/kg, 10 µg/kg i.p.) was given at 30 min before or, alternatively, at 5 min after isoprenaline (75 or 150 mg/kg s.c.). This effect was associated with a significant decrease in oxidative stress parameters and likely maintained NO system function, providing that BPC 157 interacted with eNOS and COX2 gene expression in a particular way and counteracted the noxious effect of the NOS-blocker, L-NAME [53].

### 1.7. NO

The overall NO-system significance is commonly acknowledged [36]. While malfunctions of NO-system significance are generally implicated in various disturbances, the presented findings (i.e., schizophrenia) claim both decreased and increased NO-system function in etiopathology and, thereby, controversy in therapy, either L-NAME or L-arginine [59,70,124]. This drawback can be due to only one side of the NO-system employed in the studies (i.e., decreased function by L-NAME application) while the other part remained unchecked (i.e., over-increased function by L-arginine, and vice versa) [124,125].

Thus, it can be that the general NO-system significance goes against the mentioned specific NO-agent drawbacks and against the current lack of suited NO-related therapy to specify and resolve given etiopathology [124,125]. Nevertheless, these can substantiate BPC 157 pleiotropic beneficial effects [124,125] based on the wide antagonizing potential of BPC 157 therapy on various NO-system disturbances (L-NAME-related, L-arginine-related [124,125], NO-donors-related (report in preparation)). As demonstrated, a close relationship occurred in a particular way [124,125]. Thereby, BPC 157/NO relations (i.e., as both NOS-inhibition and NOS-over-activity could be harmful, and a modulating effect applicable in either circumstance should be needed to maintain the NO-system properly balanced and adequately functioning [124,125]) can appear as the essential neurotransmitter evidence. As essential proof, we used confirmation by the exogenous application of the NO-agents given that they would mimic the NO-system corresponding to endogenous significance and effect, all as a whole, as an essential neurotransmitter evidence proof. Thereby, the application of NO-agents, NOS-blockade L-NAME, NO-over-activity (L-arginine, NOS-substrate) and their relationships (opposite or parallel activities, mutual counteraction or not), which can cover all essential points of NO-system, and the full spectrum of NO-system functions was combined with BPC 157 application to show its full efficacy on maintaining and/or reestablishing NO-system effects and functions [124,125].

Thus, there are both practical and theoretical advantages to provide the closest possible interconnections. BPC 157 application was always combined with the application of the NOS-blocker L-NAME (NOS-blockade), and NOS-substrate, L-arginine (NO-system over-activity), given alone and/or together (L-NAME (NO-system blockade), and L-arginine (NO-system-over-activity), combined L-NAME+L-arginine (NO-system immobilization). Such a triplet approach simultaneously investigated NO-system blockade/over-activity/immobilization [124,125], providing particular circumstances that required quite extensive research. Using a variety of models, NO-agents combined would either antagonize each other effect given NO-specific effect or may not antagonize each other response, or at least no antagonization to the extent of the control values; thereby, they appear to be NO-non-specific. The more models and more agents employed (NOS-blockers, NOS-substrate, combination of the NO synthase-blocker and NO-synthase-substrate) ascertains for BPC 157/NO-system the more precise relationships were defined [124,125].

This can highlight how the NO-system as a whole may work in each particular circumstance and may interact with BPC 157 or be interacted with BPC 157. There, these essential BPC 157/NO-system complex relations in a particular way could be consistently pointed out (note, “one side approach” (i.e., NO-system blockade only investigated) simple approach, most regularly used in NO-studies could be certainly less precise to determine NO-system functions as a whole) [124,125].

The summary of such triplet complex approach (L-NAME versus L-arginine versus L-NAME + L-arginine complex application) in the recent review provides a series of more than 80 distinctive targets included in the study [124]. This revealed at least six types of NO-system relations in different organs and functions. This can be a retelling of the functions of the six presentations of the NO-system, presented in the form of an interconnected network [124,125]. This particular “mapping” of the bodily NO-system by distinctive NO-agent effects and their relations (triplet complex approach) conveys that L-arginine and L-NAME may be responsible or non-responsible. They can have an opposite effect (most commonly) or parallel effect (note, this parallelism occurs with quite distinctive models, i.e., amphetamine-effect [70], miosis [91], atropine-mydriasis [91], huge magnesium over-dose [85], ischemic/reperfusion colitis [49], duodenal congestion lesions [251], cecum perforation [174]). Finally, these parallel effects can or cannot antagonize each other’s effect; thereby, particular NO-system functioning can be revealed as being NO-system specific or NO-system non-specific [124].

Centrally, such “mapping” [59,70], i.e., “L-NAME non-responsive, L-arginine-responsive” provides that counteraction by BPC 157 of the cognitive dysfunction, novel object recognition test, as a particular resembling “negative-like” symptom fully corresponds to the counteracting of the resembling “positive-like” symptoms (acute apomorphine-, chronic methamphetamine-, acute MK-801-induced effects and acute haloperidol-induced catalepsy) [59,70]. A common chain of events follows BPC 157 (antagonization), which goes over L-arginine (antagonization). L-NAME (not effective by itself) antagonizes an L-arginine beneficial effect (NO-system immobilization, L-NAME+L-arginine rats as control rats). BPC 157 co-administration restated a beneficial effect in the NO-system immobilization (the L-NAME+L-arginine+BPC 157 rats), and BPC 157 overwhelmed the effect of L-arginine as well as the effect of L-NAME and counteracted NO-system immobilization. Likewise, “L-NAME responsive, L-arginine responsive, parallel beneficial effect” NO-response might combine the recovered acute amphetamine-induced effects as the resembling “positive-like” symptom [70] and ketamine-prolonged exploration time in the ketamine-induced cognition dysfunction as the resembling “negative-like” symptom [59]. Further, NO-system immobilization was not achieved (L-NAME and L-arginine did not oppose each other’s effect), which might indicate the essential involvement of other systems as well [59,70]. Consequently, the newly revealed combined NO-system background (i.e., common therapy effect, common NO-response) [59,70] might, for BPC 157 therapy, particularly allocate matching “positive-like” and “negative-like” symptoms or indicate specific circuits between the involved brain areas, mesolimbic pathways in the “positive-like” symptoms and fronto-cortical temporal and cortico-striatal pathways in the “negative-like symptoms” [1]. Thereby, in ketamine-rat, BPC 157 therapy was able to affect all negative-like symptoms in the same way (i.e., BPC 157 counteracts cognition deficit, social withdrawal, anhedonia and has an additional anxiolytic effect), L-arginine and L-NAME could ameliorate some but worsen others [59].

All these can be its modulatory effects, interaction with several molecular pathways [8,11,75,115,116,117,118,119,120,121,122,123], in particular with NO-system [121,122,123,124,125,126,127,128,129,130] as a whole, given the BPC 157/NO-system interaction in controlling blood pressure by a particular mechanism [124,125]. BPC 157 induced the release of the NO on its own [124,125] also in the conditions where L-arginine is not working, i.e., release resistant to L-NAME, inhibiting that NO-over-release by L-arginine [124,125]. As proof of effective control and modulatory effect, BPC 157 therapy effects counteracted in the same dosage range the adverse effects of NO-system disturbances, i.e., NOS-blockade (L-NAME)-induced pro-thrombotic and hypertensive effect as well as NOS-over-activity (L-arginine)-induced anti-thrombotic and hypotensive effect [126,128], and maintained thrombocytes function [128,129,130,131] also in aggregometry and thromboelastometry studies. Likewise, the counteraction of the isoprenaline myocardial infarction using BPC 157 therapy might include a NO effect [53]. All these points support the further target’s (i.e., smooth muscle, i.e., pupil [86], or lower esophageal sphincter and pyloric sphincter [92] and urethral sphincter [100], and blood vessel [122]) sensitivity to NOS-blockade or NOS-over activity that can be distinctive. As a common point, we revealed BPC 157 beneficial effects regularly, and in these terms, likely related to particular controlling and maintaining undisturbed NO-system functions, likely applicable also to any particular circumstance. The multitude of targets can be specific given the activation of the VEGFR2-Akt-eNOS signaling pathway without the need for other known ligands or shear stress and evidenced controlling vasomotor tone by the activation of the Src-Caveolin-1-eNOS pathway [121,122]. These findings were supported by the relaxation of large vessels (ex vivo), mainly by acting on vascular endothelial cells but also on vascular smooth muscle cells, increasing NO production and migration of endothelial cells, activating the signal pathways of Src, Cav-1, and eNOS, and reducing protein–protein interaction between eNOS and Cav-1 [121,122]. The specific mechanism may also be by activating *VEGF-A*/*VEGFR1*-mediated *AKT* and *p38/MAPK* signaling pathways to upregulate downstream *eNOS* expression, which may increase the NO product synthesis, thus promote angiogenesis of gastric mucosa and also interact with endoplasmic reticulum (ER) stress. Clopidogrel increased the ER stress marker *CHOP* expression and inhibited the *eNOS* phosphorylation compared with the control group, and these changes were both reversed using BPC 157 treatment. The addition of L-NAME partly abolished the effect of BPC 157 [123]. Consistently, these should be overseen with the evidence that rather than interacting with receptors on the plasma membrane of their target cells; the gasotransmitters can cross the cell membrane and act directly on molecules inside the cell [36]. Further, as final supportive vascular evidence, there is the supportive role of BPC 157 and BPC 157 therapy in counteraction of the occlusion/occlusion-like syndromes, in general, and resolving the disturbances in particular that should be backed by the particular interaction and close connections with NO-system [40,41,42,43,47,48,49,50,51,52,53,54,55,56]. Finally, the stable gastric pentadecapeptide BPC 157/NO-system relations should consider the BPC 157 original presence in human gastric juice (not destroyed in human gastric juice more than 24 h) [1,2,3,4,5,6,7,8,9,10,11,15,27,28,29,30,33,109,124,125]. This follows its role as a cytoprotection mediator transmitting the mucosal integrity maintenance to other organs therapy [1,2,3,4,5,6,7,8,9,10,11,15,27,28,29,30,33,109,124,125] in consideration of the regular dietary taking (dietary nitrate as an important source of NO in mammals [252]), and nitrate-nitrite-NO pathway (and swallowed nitrite) resulting with high NO concentration in the stomach [253]. This can ascertain a special BPC 157/NO relation since the very beginning.

## 2. Nerve-Muscle

The particular effect on muscle integrity and functions of the BPC 157 therapy was already reviewed, and thereby, its close involvement in the course and outcome of the nerve-muscle transmission [1,3,4].

*Succinylcholine.* There, as mentioned before (see Chapter Acetylcholine), BPC 157’s counteraction/attenuation of all of the effects of depolarizing neuromuscular blocker succinylcholine applied intramuscularly (systemic and local effects; and acute, subacute, and chronic) shows a definitive rescue of disabled neuromuscular junctions (both phase I and phase II) and counteracts failure of nerve-to-muscle transmission [81]. There, the supporting argument is a rapid and sustained counteracting ability of BPC 157 in wide dose ranges (µg-ng), given intraperitoneally or per-orally before or shortly after succinylcholine administration. This includes the counteraction presented in the short term for all succinylcholine disturbances (i.e., agitation, muscle fasciculation). Likewise, this includes as well those seen in the long term (i.e., a counteracting of the ensuing systemic muscle disability [254] (i.e., paralysis) and consequent muscle damage (in the injected muscle and non-injected muscle, hyperkalemia [255] and arrhythmias [256]), with leg-contractures rapidly eliminated [81]. Further, there is otherwise increased severity of succinylcholine—applied intramuscularly [81], given the near absence of intrinsic muscle cholinesterase, an enzyme that hydrolyzes succinylcholine [257,258]. Thereby, succinylcholine injection into various muscles may regularly impair the function of these muscles long after systemic paralysis has abated [259,260]. The duration of paralysis produced by intramuscular injection is longer than that of intravenous succinylcholine application [257,258]. In addition, the BPC 157 dose-dependently counteracted the effects of the non-depolarizing neuromuscular blocker and muscle relaxant rocuronium (report in preparation). Further, we can assume that the neuromuscular junction function is a needed precondition that BPC 157 therapy (effective in rat injury, given intraperitoneally or in drinking water or topically, at the site of injury) induces the healing of the transected muscle [71], crushed muscle [72], denervated muscle [73], and myotendinous junction [74]. Note that this was performed along with the healing of the transected [135] and detached tendon and transected ligament [261,262,263], as well as bone injuries [264,265].

*The recovery of muscle dysfunctions in rats and mice with distinctive injuries. Systemic muscle disabilities.* Likely, the same innate principle can work in the recovery of muscle dysfunctions in rats and mice with distinctive injuries. The prime injuries were induced centrally (i.e., spinal cord compression [76,77], stroke [38], brain trauma [78]). The other injuries were initiated at periphery (i.e., nerve transection [79], abdominal aorta occlusion [82], tumor cachexia [11], or application of neurotoxin [69,80], distinctive muscle relaxants [81], neuroleptics [68,70], L-arginine [86], L-NAME [70,86], alcohol acute and chronic intoxication [63,64], and electrolytes disturbances [54,83,84,85]). Thus, this can be a common principle; BPC 157 therapy recovered all of these systemic muscle disabilities, peripheral and central, and the effects combined with cytoprotective capabilities (prime lesion attenuation) or neurotransmitter nerve-muscle effect clearly presented.

*Disabled heart functioning and disabled smooth muscle functioning.* Further, a point related to BPC 157/NO-system relation as well, as the BPC 157 therapy might be the recovery for the disabled heart functioning (myocardial infarction [53], heart failure [87], pulmonary hypertension [88], arrhythmias [40,41,42,47,48,49,50,51,52,53,54,55,56,60,81,89,90,91], and thrombosis prevention and reversal [40,41,42,43,47,48,49,50,51,52,53,54,55,56]). Likewise, there is the recovery of disabled smooth muscle functioning (various sphincter function recovery [86,92,93,94,95,96,97,98,99], adaptation following intestine resection, and healing following anastomosis creation [103,104]).

In conclusion, it was suggested that these findings together might provide practical realization of the multimodal muscle-axis impact able to react depending on the condition and the given agent(s) and the recovery of the muscle disturbed symptoms distinctively related to the prime injurious cause symptoms healing. This evidently ascertained the translation of the central beneficial effects to the preserved peripheral functions (i.e., muscle function) [3,4]. This was regarded as a well-functioning cytoprotection loop (brain–periphery) that might consistently occur as an implementation of the full complexities of the brain–gut axis function in BPC 157 therapy [1,3,4]. Possibly, this can be a sign of neurotransmission on its own and possible innate involvement of BPC 157 as well. Similar evidence of BPC 157 therapy might have been beneficial to the concussive brain traumas and preserved righting reflexes in the concussed mice [78]. Likewise, tail function was restored with BPC 157 therapy in rats after spinal cord compressions and tail paralysis [76,77]. An instructive example might also be the counteracted effect of the cuprizone, a neurotoxin commonly used to produce multiple-sclerosis-like lesions in rats, attenuated brain lesions, and recovered muscle functions [80].

## 3. Nerve-Nerve

To further illustrate nerve-nerve influence and possible BPC 157 significance and allocate its presence, we raised several arguments.

*Healing of transected rat sciatic nerve.* First, we used the consistent failure of spontaneous healing of transected rat sciatic nerve in anastomosed nerve [79]. Consistently, we also used non-anastomosed nerves (7 mm nerve segment resected). These results with the evidence that BPC 157 markedly improved rat sciatic nerve healing. This was evidenced clinically (autotomy absent [79]; autotomy reflects chronic neuropathic pain, neuroma at the proximal nerve stump, regenerative nerve sprouts growing into all directions; counteracted pathophysiological impulses and increased spontaneous activity in the spinal dorsal horn at the segments of projection of the injured nerve [266]). Likewise, this was consistently evidenced histomorphometrically, electrophysiologically (increased motor action potentials), and functionally (walking recovery (sciatic functional index (SFI)) [79]. BPC 157 effect was quite persistent, combining both local and systemic effects [79]. Immediately after nerve anastomosis creation, BPC 157 was applied intraperitoneally, intragastrically, or locally at the site of anastomosis. To replace the dissected nerve segment, BPC 157 was applied directly into the tube to fill the gap between the nerve stumps [79]. Further evidence of the innate effect on neurons goes with the mentioned BPC 157 application, which increased both the survival rate of cultured enteric neurons and the proliferation of cultured enteric glial cells (EGCs) [119]. For somatosensory neurons, counteraction of the several gastric lesions of distinctive background (i.e., intragastric alcohol, indomethacin, restraint stress) can locate BPC 157 within somatosensory neurons functions as BPC 157 collaborates with capsaicin somatosensory neurons in a particular way [267]. BPC 157 therapy promoted the beneficial effect of low-dose capsaicin (i.e., micrograms/kg, transient excitation). Contrarily, BPC 157 therapy counteracted the damaging effect of systemic administration of high doses of capsaicin (milligrams per kilogram range, long-lasting damage of somatosensory neurons, more when given in newborn animals). Thus, the prime beneficial function of somatosensory neurons is emphasized. This is combined with the recovery of defunctionalized capsaicin-sensitive primary afferent neurons. This beneficial effect of BPC 157 therapy also occurs in adult rats when capsaicin is given to newborn animals [267].

*Various brain disturbances and spinal cord injuries.* Further, using the same regimen, BPC 157 cured various brain disturbances and spinal cord injuries [1]. Thereby, the various brain areas’ consistent healing and recovered functions can be an indication of the recovered functions of the involved neurotransmitters function, and BPC 157 function on its own. Illustratively, therapy in the reperfusion after 20 min-bilateral clamping of the common carotid arteries [38] showed the effects of BPC 157 [38] in parallel with those that might occur in stroke in rats. The therapy counteracted both early and delayed neural hippocampal damage, achieving full functional recovery (Morris water maze test, inclined beam-walking test, lateral push test) [38], as assessed at 24 h and 72 h of the reperfusion. Such neurotransmitter generalization can be in a wide therapy range previously seen in terms of cytoprotection and evidenced implementation with the pleiotropic agent’s beneficial activity [1]. Such counteraction of encephalopathies in terms of possible ubiquitous neurotransmitter role (i.e., the exogenous effects and endogenous presence to verify endogenous presence and significance) includes the distinctive noxious agents, specifically affecting all brain structures (and consequently neurotransmitter disabilities) with the areas most affected by lesions most presented. These were, i.e., in the cerebellum (ibuprofen [107], paracetamol [61]), the cerebral cortex (diclofenac [106,108], celecoxib [105]), the frontoparietal cortex (concussive brain trauma) [78], the hippocampus and the cerebral cortex (insulin) [62], and the parietal neocortex and hippocampus (cuprizone [80]) (note, cuprizone application [80] was an extremely high regimen highly over those commonly applied to mimic multiple sclerosis in rats [268,269]).

Therefore, this can be a specific therapy depending on the particular brain injury. This can be stated given the particular counteracting effect on convulsions (i.e., insulin hypoglycemic convulsions [62], and paracetamol over-dose-convulsions, progressive hepatic encephalopathy with generalized convulsions [61]), the improved conscious/unconscious/death ratio in trauma brain injury mice [78], attenuated/counteracted cerebellar ataxia, fore and hind limbs disabilities in cuprizone rats [80], and counteracted sedation/unconsciousness (diclofenac [108], ibuprofen [107]). Likewise, counteracting all peripheral lesions, thought to be a part of the ongoing cytoprotection along with the cytoprotective agent’s application, can be consequent to particular neurotransmission toward all affected targets. Counteraction of the huge liver lesions (particular lesions presentation depending on the paracetamol [61], diclofenac, ibuprofen (i.e., hepatomegaly) [107], celecoxib [105], and insulin (i.e., calcification) [62]) and gastrointestinal lesions (i.e., diclofenac [108], ibuprofen [107], celecoxib [105], insulin [62]) can be a proof of the concept. Illustrating the complexity of the events that should be involved, for the counteracted effects of insulin-over dose [62], this can be the recovery of both insulin pathways (i.e., prominent counteraction of calcification of liver blood vessels [62] requires both insulin pathways inhibited [270,271]). Acute brain refueling [62] can be from the peripheral source (counteracted insulin seizures [62]) and the recovered activity in previously hypoglycemia-silenced brain regions such as the cortex, hippocampus, and amygdala [270].

*Occlusion/occlusion-like syndromes.* From the viewpoint of neurotransmitters and neurotransmission for the brain, cerebral, cerebellar, hypothalamus, and hippocampus lesions, and cytoprotection as innate background for the interconnected pleiotropic beneficial effect, there is growing evidence that BPC 157 therapy can counteract the occlusion/occlusion-like syndromes [40,41,42,43,47,48,49,50,51,52,53,54,55,56]. This beneficial action is peripherally and centrally, as well as centrally and peripherally, in rats with occluded major vessels peripherally and centrally or which underwent similar noxious procedures and various agents’ applications that all severely affect endothelium. Commonly, these might be three body cavities interconnected and rapidly communicating with each other, and disturbances rapidly transmitted through the venous system unless rapidly upgraded by the given therapy (activation of collateral pathways, azygos vein direct blood flow delivery). Additionally, commonly, counteraction includes the lesions in the brain (intracerebral, intraventricular hemorrhage), heart (myocardial infarction, congestion, arrhythmias), lung (hemorrhage), congestion in the liver and kidney, and gastrointestinal lesions, progressing thrombosis, peripherally and centrally, attenuation/elimination of intracerebral (superior sagittal sinus), portal and caval hypertension, aortal hypotension occurred as the counteraction of trapped blood volume and vascular and multiorgan failure. Thereby, it can be, in general, a vascular recovery depending on the given injury [40,41,42,43,47,48,49,50,51,52,53,54,55,56]. Thus, an illustration is the above-mentioned neuroleptic-domperidone-amphetamine occlusion/occlusion-like syndrome [54], in particular (i.e., haloperidol, fluphenazine, clozapine, risperidone, olanzapine, quetiapine, aripiprazole, domperidone, amphetamine, and combined amphetamine and haloperidol all produced similar syndrome). It can be that BPC 157 therapy commonly indicated vascular recovery as an additional route leading to or from the specific receptors for the agonists and antagonists rather than the receptors themselves [54]. Note, considering the BPC therapy of spinal cord injury (compression, tail paralysis recovered), there is in the spinal cord, upon BPC 157 therapy, in rats, which had spinal cord compression, the occurrence of rapid regression of the spinal cord hematoma upon therapy [76,77]. This occurred when therapy was given after the injury at 10 min and for the first time after 4 days following the injury [76,77].

## 4. Channels

This can be a particular effect of the BPC 157 therapy on channel activity [60,83,84,85,89]. This was shown in HEK 293 cells, known to have voltage-gated K^+^ [272,273], Na^+^ [274], and Ca^2+^ [275] channels, and with different local anesthetics applications (i.e., bupivacaine [89], lidocaine [60], tetracaine [276], and oxibuprocaine [276]). Generally, if the receptors are ligand-gated ion channels, a conformational change opens the ion channels, which leads to a flow of ions across the cell membrane resulting in either a depolarization, for an excitatory receptor response, or a hyperpolarization, for an inhibitory response [277].

In HEK 293 cells in hyperkalemic conditions [83], BPC 157 inhibits potassium channels and, therefore, induces a reduction in depolarization under hyperkalemic conditions. BPC 157 alone depolarized HEK293 cells; the same depolarization was blocked by the nonspecific potassium channel inhibitor BaCl_2_ [83]. In addition, in the presence of BPC 157 (1 µm), depolarization induced by bupivacaine [89], lidocaine [60], and magnesium [85] was inhibited. The same BPC 157 regimen abolished hyperpolarizations of HEK293 cells during hypokalemic conditions [84]. Such direct effect can ascertain channel functioning in disturbed electrolyte circumstances (i.e., hyperkalemic [83] and hypokalemic [84] or hypermagnesemic [85] condition) and counteraction of channel disturbances that were induced using the agents that otherwise block channel function. This goes along with full in vivo effect, counteraction of the hyperkalemia harmfulness as well as hypokalemia harmfulness (including pressure disturbances, arrhythmias, sphincter dysfunction, and fatality related to hyperkalemia or hypokalemia) [83,84]. Likewise, in hypermagnesemia, BPC 157 counteracts adverse effects (i.e., severe muscle weakness and prostration, decreased muscle fibers, nerve damage, and edema in various brain areas, with the most prominent damage in the cerebral cortex) [85]. Likewise, this goes along with counteraction/attenuation of bupivacaine (i.e., arrhythmias, fatal outcome) [89], lidocaine (i.e., local anesthesia via an intraplantar application and axillary and spinal (L4–L5) intrathecal block, arrhythmias, convulsions) [60], tetracaine (i.e., dry eye) [276], and oxibuprocaine (i.e., dry eye) [276] as a whole. Thus, the counteracting effect of BPC 157 therapy should comprise the primary mechanism of action. This implies sodium channel inhibition, a blockade of voltage-gated sodium channels (VGSCs) leading to a reversible block of action potential propagation [278], and reduced permeability of cell membranes, thereby blocking depolarization and preventing the conduction of the electrical impulse through which pain occurs [279].

Moreover, counteraction of the lidocaine effect [60] (i.e., lidocaine occupied receptors on the sodium channel in its open/active (phase 0) and inactivated/refractory (phase 2) states [280]) can suggest that, as lidocaine, BPC 157 itself can have a high affinity for its receptors during both phases.

The counteracted effects of lidocaine and bupivacaine [60,89] can suggest an extended effect even beyond the prime local anesthetic effect. Illustratively, BPC 157 counteracted lidocaine convulsions [60], and thereby, likely also lidocaine’s effects on the central nervous system (i.e., inhibiting nicotinic acetylcholine receptors, inhibiting presynaptic calcium channels in the dorsal root ganglion, inhibiting opioid receptors, inhibiting of neurite growth, inhibiting muscarinic cholinergic receptors, and preventing substance P from binding to natural killer (NK) cell receptors) [278,281,282,283].

Likewise, with BPC 157 therapy, counteraction of bupivacaine arrhythmias and fatality [89] should also include, in addition to blocking Na^+^ channels, the bupivacaine activity of many other channels, including NMDA receptors. Importantly, bupivacaine inhibits NMDA receptor-mediated synaptic transmission in the dorsal horn of the spinal cord, an area critically involved in central sensitization [284]. 

In summary, these findings [60,83,84,85,89,276] would approach the channel functioning and the BPC 157 counteracting effect, and thereby, in particular, BPC 157 presentation to the essential chain of the events following the application of local anesthetic [285,286]. Alternatively, or in addition, this may be its essential penetration of both the epineurium and neuronal membrane during the action for the time that a local anesthetic remains near neural fibers. Both of these possibilities have fair support. As mentioned, BPC 157 may interfere with the early events (i.e., immediate application after lidocaine) [60]. Likewise, it counteracted the already established late lidocaine-induced effects (i.e., given later after lidocaine, with a 10 min delay) [60] (i.e., the local sequestration of highly lipid-soluble anesthetics may allow for continual release to the neuronal membranes, prolonging duration [285]).

## 5. Receptors

Conceptually, neurotransmitter activity, as the basis for the cytoprotective effects of the implemented agents, was not in the implementation of the cytoprotection concept since seminal studies [16,17,18,19,20,21]. On the other hand, commonly, the activation of receptors should be the only direct action of a neurotransmitter [12,13,14].

Nevertheless, based on the described findings (see Chapters Dopamine, Serotonin, Glutamate, GABA, Noradrenaline/Adrenaline, Acetylcholine, and NO), the activation of receptors in BPC 157/neurotransmitters/neurotransmission relations has to be a particular one and concerns particular ability, multifacetedness, given distinctive disturbances, each particularly counteracted. The proof can be the equal counteracting effect on the consequences of various receptors blockade as well as on the consequences of the various receptors activation and disturbances such as tolerance or hypersensitivity (see Chapters Dopamine, Serotonin, Glutamate, GABA, Noradrenaline/Adrenaline, Acetylcholine, and NO) (see, Table 1, Table 2, Table 3, Table 4 and Table 5 and Figure 2). There is an intriguing common highlight considering the number of the receptors and disturbances involved and attenuated/counteracted but fully along with BPC 157 general cytoprotection background, and thereby the innate (cytoprotective) wide ability to counteract various noxious events [1,2,3,4,5,6,7,8,9,10,11,15,27,28,29,30,33,109,124,125] (see, Figure 1). This could be the role of maintaining or reestablishing normal circumstances and functioning. This can be a particular structure of such potential, allowing counteraction within the same dose range of quite distinctive but specific effects directly allocated with receptors. This is the counteracting effect on distinctive dopamine disturbances (counteracted adverse effects of dopamine receptors blockers [68,70]; counteracted the effects of dopamine agonist, direct and indirect [70,152,153]), and the counteracting effect on distinctive serotonin disturbances [65,66,67] (selective release of the brain serotonin [66] and counteracted serotonin syndrome as a whole [67]). Such particular wide potential also occurred with glutamate disturbances [59,70] (i.e., counteracted ketamine-cognition dysfunction,-social withdrawal, and -anhedonia versus exerted additional anxiolytic effect in ketamine rats [59]; counteracted locomotion, stereotyped sniffing, and ataxia as a sign of positive-like symptoms of schizophrenia [70] induced using MK-801 (dizocilpine)). The same goes for GABA-disturbances (counteracted thiopental anesthesia (GABAA receptor currents increased) [218]; counteracted both non-competitive and competitive GABAA receptor antagonists) [15,58]. This dual counteracting potential occurred also with the acetylcholine-disturbances (nicotine receptor agonist (succinylcholine) [81] and antagonist (rocuronium) (report in preparation) both counteracted; counteracted muscarinic receptor agonist (pilocarpine) and antagonist (atropine)) [91,244] and adrenaline/noradrenaline-disturbances (counteracted both isoprenaline (beta agonist) and sotalol (beta antagonist) effects) [52,53] (see, Table 1, Table 2, Table 3, Table 4 and Table 5 and Figure 2). Similar dual counteracting potential exists with NO-system (counteracted were the adverse effects of NO-system blockade and adverse effects of NO-system over-stimulation) [124,125]. BPC 157 markedly decreased the eNOS/Cav-1 binding and decreased Cav-1 binding, released the eNOS, and subsequently enhanced the activation of eNOS [121]. Finally, counteracted were the distinctive chains of events leading to receptor disturbances that occurred as diazepam-tolerance/withdrawal, -physical dependence [57], haloperidol dopamine receptor hypersensitivity [152], and amphetamine reverse tolerance [153] (see Table 1, Table 2, Table 3, Table 4 and Table 5 and Figure 2).

Therefore, there must be common endogenous mechanisms beyond and outside of the mentioned systems: dopamine, serotonin, glutamate, GABA, adrenergic/noradrenergic system, acetylcholine, and NO-systems. In support of a special system, a study (Pharmacological receptor binding affinity of PL-10.1. Istituto di Ricerche Biomediche “Antoine Marxer” RBM S.p.A., RBM Exp. 950020; 1995.) has shown that BPC 157 did not display any pharmacological affinity for any of the receptor systems studied. The interaction of BPC 157 (PL 14736) with adrenergic α- and β-1 and two receptors, with cholinergic muscarinic mr, m_2_- and m_3_-, with histamine Hr and H_2_-receptors, with serotonin 5-HT_1_-^.^ and 5-HT_2_, with dopamine D_1_- and D_2_, and with A_1_ and A_2_-adenosine receptors was investigated. However, such a wide range of even opposite disturbances that can be all attenuated/counteracted, given a general counteracting potential, can match with the original cytoprotection concept perception holding the equal cell protection (necrosis prevention) against a variety of different agents (originally used absolute alcohol, boiling water, strong base, and strong acid claimed for common endogenous mechanisms). Consistently, given the close BPC 157/NO-system relations (see Chapter NO), these should be overseen with the evidence that the gasotransmitters can cross the cell membrane and act directly on molecules inside the cell [36], and thereby, particularly interacting with receptors on the plasma membrane of their target cells can be envisaged.

On the other hand, the defining BPC 157-wound healing as a part of the cytoprotection endothelium effect attracted BPC 157/vascular endothelial growth factor (VGEF) relation (signaling proteins involved in vasculogenesis and angiogenesis) [6,10]. As consistent evidence, BPC 157 healing (transected, crushed, denervated muscle, myotendinous junction [71,72,73,74,75], tendon [135], osteotendinous junction, ligament, bone [261,262,263,264,265] healing) resolved “wounded phenotype”, all chain of events as a whole (i.e., bone fractured, skin torn, blood vessels ruptured, ligaments or muscles damaged) [6,10]. Likewise, the healing implies resolution as a whole of several temporally coordinated processes driven by locally released mediators toward tissue repair and the coagulation cascade as well [287]. Note, BPC 157 therapy can attenuate/eliminate both progressing thrombosis and hemorrhage in occlusion/occlusion-like syndromes [40,41,42,43,47,48,49,50,51,52,53,54,55,56] and attenuate bleeding following amputation [128,129,130], organ perforation [49,173,174], anticoagulants [128,129,130], and antiplatelet agent [128,129,130] application, and resolved even advanced Virchow triad circumstances [40,41,42,43,47,48,49,50,51,52,53,54,55,56]. The increased expressions of VEGF, CD34, and FVIII were shifted toward the left in crushed muscle, transected muscle, and transected tendon [288] along with improved angiogenesis along with improved healing, as particular effect (note, BPC 157 cures corneal ulcer and maintains corneal transparency [113], and inhibits VEGF effect in human melanoma cell line [289]). This can be performed by controlling the VGEF system as well.

Recently, BPC 157 treatment increased the expression of VEGF-A in alkali-burn skin wounds of rats [116]. Further, a study focused on the angiogenic effect and counteraction of limb ischemia demonstrated that BPC 157 itself not only increased the VEGFR2 expression in vascular endothelial cells several hours after treatment but also immediately triggered the internalization of VEGFR2 within minutes [122]. Subsequently, it activated the phosphorylation of VEGFR2, Akt, and eNOS signal pathway without the need for other known ligands or shear stress. The suppression of BPC 157-induced VEGFR2, Akt, and eNOS activation and tube formation by dynasore, an inhibitor of endocytosis, further supported the importance of VEGFR2 internalization underlying the mechanism of BPC 157 effect [122].

A study resolving the counteraction of clopidogrel and disturbed angiogenesis by BPC 157 application can be particularly indicative. Clopidogrel down-regulated the *VEGF-A* and *VEGFR1*, subsequently inactivated *AKT* signaling pathway and induced phosphorylation of *p38/MAPK* and *ERK/MAPK*; furthermore, BPC 157 could significantly up-regulate the *VEGF-A* and *VEGFR1*, paralleled by the phosphorylation of *AKT*, and inactivation of *p38/MAPK* and *ERK/MAPK* signaling pathways. More importantly, the NO synthase blocker L-NAME can reverse these effects of *AKT* and *p38/MAPK* signaling pathways induced using BPC 157 but cause no changes in *ERK/MAPK* [123]. The evidence that BPC 157 can have a particular effect on *ERK/MAPK* [123] suggests that it can affect the *ERK/MAPK* pathway as a chain of proteins in the cell that communicates a signal from a receptor on the surface of the cell to the DNA in the nucleus of the cell [290].

Thereby, it can be that BPC 157 can particularly interfere with the mentioned neurotransmitter systems via particular influence on the VGEF-system, given that VEGF, in addition to the NO-system [121,122,123], can interact with dopamine, serotonin, glutamate, GABA, acetylcholine, and adrenaline/noradrenaline. There is evidence that VGEF can be particularly involved in schizophrenia [291,292,293,294], given abnormal vascularization and VEGF acting as an angiogenic and neurotrophic factor involved in the regulation of cerebral blood volume and flow in schizophrenia [294]. Note, the regulation of the brain blood flow was ascribed to BPC 157 therapy in occlusion/occlusion-like syndrome (including also in amphetamine rats) given rapid decrease in the brain swelling and counteracted intracranial hypertension along with activation of the collateral rescuing pathways immediately upon BPC 157 therapy [40,41,42,43,47,48,49,50,51,52,53,54,55,56]. The levels of VEGF were significantly higher in medicated multiple-episode schizophrenia [292]. The high VEGF levels, associated with better responses to antipsychotics, might be predictive of the use of first-generation antipsychotic drugs, whereas low VEGF levels, expression of resistance to therapy, might be predictive of the use of second-generation antipsychotic drugs) [294].

In addition, VEGF-A can control the expression of dopamine D2 receptors [295]. VEGF-induced antidepressant effects involve modulation of norepinephrine and serotonin systems [296]. VEGF is induced using multiple classes of antidepressants at time points consistent with the induction of cell proliferation and therapeutic action of these treatments [297,298]. VEGF, GABA, and glutamate signaling can be particularly connected [299]. VEGF modulates the GABA and glutamate neurotransmission systems and suppresses epileptic activity in an experimental model of temporal lobe epilepsy [300]. Moreover, VEGF enhances GABA synaptic transmission during spinal cord development [301]. The endogenous-acetylcholine-induced VEGF expression in neurons and astrocytes [302], the VEGF signaling system is in the rescuing effect of acetylcholine on NMDA-induced long-lasting hippocampal cell damage [303]. VEGF promotes vascular sympathetic innervation and appears as one of the modulators of vascular sympathetic innervation [304,305].

In addition, as demonstrated in a study of BPC 157/tendon-healing relations [117,118], in tendon fibroblasts, BPC 157 increased the expressions of growth hormone receptors at both the mRNA and protein levels given the persistent promoting effect on the expression of growth hormone receptor as more significant up to three days after the treatment. Therefore, a similar relation to the BPC 157/VEGF relation can be made for the BPC 157/growth hormone relation, given the pleiotropic involvement of the growth hormone. Illustratively, growth hormone is implicated in the development of schizophrenia [306], interconnected with glutamate [307,308], GABA [309], dopamine [310], serotonin [311], catecholamines, and acetylcholine [312,313,314].

Thus, it can be that the pleiotropic effect of BPC 157 therapy can be at least partly due to the activation of these receptors and can perceive the direct action of a neurotransmitter.

## 6. Conclusions

Although many points remain to be further shown or explained, particularly due to the use of the animal models and their general translational significance, this review attempts to resolve the shortage that the pleiotropic beneficial effects of BPC 157 may not fulfill the general standard neurotransmitter criteria, in classic terms. We attempt to substitute the lack of direct conclusive evidence (i.e., a chemical produced within the neuron or present in it as a precursor molecule, released eliciting a response on the receptor on the target cells on neurons and being removed from the site of action once its signaling role is complete). This can be a network of interconnected evidence previously envisaged in the implementation of the cytoprotection effects (see Figure 1). Thus, that provides consistent beneficial particular evidence that BPC 157 therapy can counteract dopamine, serotonin, glutamate, GABA, adrenalin/noradrenalin, acetylcholine, and NO-system disturbances (see Table 1, Table 2, Table 3, Table 4 and Table 5 and Figure 2). This specifically includes counteraction of those disturbances related to their receptors, which are both blockade and over-activity, destruction, depletion, tolerance, sensitization, and channel disturbance counteraction. Furthermore, close BPC 157/NO-system relations with the gasotransmitters crossing the cell membrane and acting directly on molecules inside the cell may envisage particular interactions with receptors on the plasma membrane of their target cells.

Likewise, BPC 157 can activate particular receptors (i.e., VGEF and growth hormone) (note that BPC 157 along with NO-system can control VEGF activity (organizing angiogenesis in healing, counteracting tumor promoting effect)). Together, these findings can claim possible relations with neurotransmitters’ activity. Further, it can be that BPC 157 therapy commonly indicated vascular recovery (activation of the collateral rescuing pathways, i.e., azygos vein direct flow delivery, to reestablish the reorganized blood flow noted in the counteraction of occlusion/occlusion-like syndromes) as an additional route leading to or from the specific receptors for the agonists and antagonists rather than the receptors themselves. Such cytoprotective evidence (i.e., BPC 157 stable pentadecapeptide native and stable in human gastric juice can be released into circulation as a cytoprotective mediator, sent to distant organs) to explain the particular aspects of the stable gastric pentadecapeptide BPC 157 pleiotropic beneficial activity as a part of its cytoprotective (organoprotective) activity, and neurotransmitter’s activity as well. Conceptually, along with its endogenous presence, such a wide range of the even opposite disturbances that can be all attenuated/counteracted, nerve-muscle relation in various muscle disturbances counteraction, and nerve-nerve relation in various encephalopathies counteraction, exemplified specifically BPC 157 therapy application. Its µg-ng dose range applied alone, without carrier addition, application including also via per-oral route, clinical evidence, and toxicology without LD1, can ascertain practical applicability. In the end, this can hold a general counteracting potential and contributing innate neurotransmitter potential, matching with the original cytoprotection concept perception holding equal cell protection (necrosis prevention) against a variety of different agents, thus, a pleiotropic beneficial effects that should be obtained with cytoprotective agents’ application that could also have neurotransmitter background as well.

## Figures and Tables

**Table 2:** A restorative dimension of the BPC 157 therapy can react with the serotonin system depending on the condition [65,66,67].

Effect	Specification	Ref.
Antidepressant effect (Porsolt’s test, open field)	BPC 157 therapy (Porsolt’s test, chronic stress, reduced duration of immobility) overwhelmed the effect of imipramine.	[65]
The regional serotonin synthesis (using alpha-[14C]methyl-L-tryptophan (α-MTrp) autoradiographic measurements) in the brain following peripheral (intraperitoneal) BPC 157 administration	There was an overall decrease in brain serotonin synthesis following acute treatment, which provided an overall increase in synthesis in chronically treated rats. In acute treatment, a significant decrease in the globus pallidus, dorsal and ventral hippocampus, dorsal thalamus, lateral geniculate body, and hypothalamus. Contrarily, the synthesis significantly increased in the medial anterior olfactory nucleus and substantia nigra reticulate. In chronic treatment, a significant decrease observed in the dorsal raphe is along with increases in the substantia nigra, the lateral caudate, and the accumbens nucleus. This can likely point to a particular serotonergic response that is timely related to BPC 157 applications. The substantia nigra’s (compacta and reticulata) structure (given serotonin synthesis significantly increased following both acute and chronic treatments) occurred as a particular point of pentadecapeptide BPC 157.	[67]
Counteracting potential of BPC 157 therapy on serotonin syndrome	BPC 157 therapy counteracted serotonin syndrome initiation (i.e., counteracted pargyline effect). Then, in particular, BPC 157 counteracted the full serotonin syndrome crisis (attenuated the adverse effect of the subsequent L-tryptophan application). This effect may have been a particular effect, as BPC 157 counteracted each part of the serotonin syndrome presentations, and then, BPC 157 therapy might fully counteract serotonin syndrome [66] as a whole. Both temperature and behavioral changes in all these experiments were counteracted.	[66]
Inhibited release of enteric serotonin	Inhibited release of enteric serotonin and inhibited intestinal motility, the increased survival rate of cultured enteric neurons, and the increased proliferation of cultured enteric glial cells (EGCs) by BPC 157 application.	[119]
BPC 157 maintains platelet function in a particular way	Given in occlusion/occlusion-like syndromes, BPC 157 therapy eliminated/annihilated hemorrhage (i.e., brain, lung) and thrombosis, in particular of consideration of pulmonary embolism [87] and evidently, reversed already advanced Virchow triad circumstances [40,41,42,43,47,48,49,50,51,52,53,54,55,56]. In addition, there was the specifically maintained thrombocytes function (i.e., the opposed L-NAME-pro-thrombotic effect, opposed L-arginine-anti-thrombotic effect) [128], given the coagulation pathways not affected as also demonstrated in aggregometry and thromboelastometry studies [128,129,130], and counteracted prolonged bleeding following anticoagulants (heparin, warfarin) and anti-platelet agents (aspirin, clopidogrel, cilostazol) [126,127,128], organ perforation [49,173,174] or amputation of tail or foot [128,129,130].	[126,127,128,129,130]
In conclusion, there is a restorative dimension of the BPC 157 therapy that can react with the serotonin system depending on the condition [65,66,67,119,126,127,128,129,130].	The restorative dimension of the BPC 157 therapy, and thereby BPC 157 activity over the serotonin system, as a likely neurotransmitter of its own, can be based on the following consistent evidence providing a wide range of influence on various, even opposite, activities [1]. The arguments are its particular combination, such as antidepressant effect (Porsolt’s test, open field) [65] and region-specific influences on brain serotonin synthesis in rats given a particular increase in the substantia nigra [67] vs. counteraction of the serotonin syndrome as a whole [66]. Further, there is a reduction in enteric serotonin concentration, attenuated intestinal motility, increased survival of cultured enteric neurons, the proliferation of cultured EGCs [119], and a particular effect on maintaining platelets function [126,127,128,129,130].	

**Table 3:** A restorative dimension of the BPC 157 therapy can react with the glutamate system depending on the condition [59,70].

Effect	Specification	Ref.
BPC 157 counteracted the effect of MK-801, a non-competitive antagonist of the NMDA receptor application in rats.	BPC 157 counteracted the effect of MK-801 locomotion, stereotyped sniffing, and ataxia as a sign of positive-like symptoms of schizophrenia [70].	[70]
BPC 157 counteracted negative-like schizophrenia symptoms in rats	BPC 157 counteracted negative-like schizophrenia symptoms, ketamine-cognition dysfunction, social withdrawal, and anhedonia and exerted additional anxiolytic effects in rats. There was a distinctive ketamine dosage range, but all were counteracted using the same dosage range of BPC 157. The significance of such counteraction is further established by the additional application of NO-agents. Each of the negative-like symptoms differs from each other given their different responsibility to L-NAME and L-arginine given alone or together.	[59]
In conclusion, there is a restorative dimension of the BPC 157 therapy. It is evident that it can react with the glutamate system depending on the condition.	BPC 157/glutamate relation goes as a restorative dimension (i.e., neurotransmitter of its own over glutamate system) with the evidence that BPC 157 counteracted MK-801 positive-like symptoms of schizophrenia [70] and ketamine negative-like schizophrenia symptoms, ketamine-cognition dysfunction, social withdrawal, and anhedonia, and exerted additional anxiolytic effects in rats [59]	

**Table 4:** A restorative dimension of the BPC 157 therapy can react with the GABA system depending on the condition [15,57,58,59,63,64,218].

Effect	Specification	Ref.
BPC 157 has a particular anxiolytic effect on its own	BPC 157 has a particular anxiolytic effect in light/dark and shock probe/burying tests.	[57]
BPC 157 has a particular anxiolytic effect on its own	BPC 157 has a particular additional anxiolytic effect in ketamine rats.	[59]
BPC 157 coadministration in chronically treated diazepam mice counteracts diazepam tolerance and withdrawal, postpones physical dependence, and prolongs residual diazepam anticonvulsive activity.	BPC 157 therapy also, on its own, counteracts isoniazid (GABA synthesis inhibitor)-, picrotoxin (non-completive channel blocker for GABAA receptors (GABAARs) chloride channels)-convulsions.	[58]
BPC 157 therapy counteracts bicuculline convulsions.	BPC 157 therapy counteracts bicuculline (completive antagonists of GABAARs)-convulsions.	[15]
BPC 157 therapy counteracts in rats the anesthetic effect of thiopental [218]	BPC 157 in doses of 10 ng/kg and 10 µg/kg, respectively, caused significant counteraction of loss of righting reflex produced by thiopental with a parallel shift of the dose–response curve for thiopental to the right. Illustratively, BPC 157 therapy also counteracts the effect of L-NAME, which increases the thiopental loss of the righting reflex seven times.	[218]
BPC 157 therapy exhibited the counteraction of both acute and chronic alcohol effects as a highlight of the BPC 157 particular potential consistently evidenced in mice that were either acutely intoxicated or physically dependent on alcohol [63,64].	BPC 157 intraperitoneally or intragastrically strongly prevented and reversed the effects of acute intoxication (i.e., quickly produced and sustained anesthesia, hypothermia, increased ethanol blood values, 25% fatality, 90 min assessment period) when given before or after ethanol, and none of the mice died. When given after abrupt cessation of chronic ethanol (at 0 or 3 h withdrawal time), it attenuated withdrawal and handling induced withdrawal seizures.	[63,64]
Following acute absolute alcohol intragastric administration, BPC 157 therapy attenuated/eliminated the alcohol-occlusion/occlusion-like syndrome as a whole, major vascular and multiorgan failure [56], as described above [40,41,42,43,47,48,49,50,51,52,53,54,55,56].	Intracranial, portal, and caval hypertension and aortal hypotension, lesions and hemorrhage in the brain, heart, lung, liver, and kidney, and thrombosis peripherally and centrally were all counteracted along with counteraction of the prime major stomach alcohol lesion. The therapy effect was ascribed to the counteraction of the congestion of major vessels and particularly to activation of the rescuing collaterals, i.e., azygos vein direct blood flow delivery. BPC 157 therapy effectiveness illustrates that brain swelling instantly decreases. A fall of intracranial hypertension occurs immediately upon BPC 157 therapy. The therapy effect was ascribed to the activation of the rescuing collaterals, i.e., azygos vein direct blood flow delivery, and to the counteraction of the congestion of major vessels that occurred instantly.	[56]
In conclusion, there is a restorative dimension of the BPC 157 therapy. It is evident that it can react with the GABA system depending on the condition.	The restorative dimension of the BPC 157 therapy, and thereby BPC 157 activity over the GABA system, as a likely neurotransmitter of its own, can be based on the following consistent evidence providing a wide range of influence on various, even opposite, activities [1]. The anxiolytic effect on its own (light/dark, shock probe/burying [57], ketamine [59]) of BPC 157 therapy is a particular effect. In support, in diazepam tolerance/withdrawal and physical dependence/withdrawal studies [58], BPC 157 coadministration in chronically treated diazepam mice counteracts diazepam tolerance and withdrawal, postpones physical dependence, and prolongs residual diazepam anticonvulsive activity [58]. BPC 157 therapy also, on its own, counteracts isoniazid (GABA synthesis inhibitor)-, picrotoxin (non-completive channel blocker for GABAA receptors (GABAARs) chloride channels)- [58], and bicuculline (completive antagonists of GABAARs)-convulsions [15]. Finally, BPC 157 therapy counteracts the anesthetic effect of thiopental in rats [218], a prototype member of the barbiturate class of drugs. BPC 157 therapy exhibited the counteraction of both acute and chronic alcohol effects [63,64]. Following acute absolute alcohol intragastric administration, BPC 157 therapy attenuated/eliminated the alcohol-occlusion/occlusion-like syndrome as a whole, major vascular and multiorgan failure [56].	

**Table 5:** A restorative dimension of the BPC 157 therapy can react with the acetylcholine system depending on the condition [81,86,244].

Effect	Specification	Ref.
BPC 157 exhibited counteraction of the succinylcholine and rocuronium (report in preparation).	In succinylcholine-rats, BPC 157 counteracted agitation before muscle disability, numerous twitches before complete loss of muscle tone, motionless prostration, and, subsequently, a painful reaction (violent screaming upon light touch). BPC 157 dose-dependently counteracted the effect of rocuronium (report in preparation).	[81]
BPC 157 counteracted the effect of muscarinic receptor agonist pilocarpine.	Additionally, BPC 157 counteracted the effect of muscarinic receptor agonist pilocarpine, miosis (and subsequent mydriasis in rats due to muscle disability), hypersalivation, and convulsions.	[244]
BPC 157 counteracted prototypical muscarinic receptor antagonist atropine, and mydriasis in rats and guinea pigs.	An equal counteracting effect following local, intragastric, and intraperitoneal application is consistent with an overall effect on acetylcholine system function. These effects appear to be NO-system related.	[86]
In conclusion, there is a restorative dimension of the BPC 157 therapy. It is evident that it can react with the acetylcholine system depending on the condition.	BPC 157/acetylcholine relation principle can be a particular way with the restorative dimension of the BPC 157 therapy, and thereby, BPC 157 activity over the acetylcholine system, as a likely neurotransmitter of its own. This can be based on the following consistent evidence providing a wide range of influence on various, even opposite, activities [1], noted in particular with BPC 157 counteraction of the succinylcholine [81], rocuronium (report in preparation), pilocarpine [244] and atropine [86] effects.	
